# Supplementary material for: Tuning flavin environment to detect and control light-induced conformational switching in Drosophila cryptochrome
Source: Commun Biol. 2021 Feb 26;4:249. doi: 10.1038/s42003-021-01766-2 (PMC7910608; doi:10.1038/s42003-021-01766-2)
Supplement: Supplementary file 3 — Description of Additional Supplementary Files [file 42003_2021_1766_MOESM3_ESM.pdf]

Description of Additional Supplementary Files

File name: Supplementary Data 1
Description: Source data for the following figures.

Source Fig2. UV-Vis and ENDOR spectroscopy of dCRY WT and L405/C416N variants. Col. A-E contains the UV-Vis data while Col. G-I contains the ENDOR data. The column header contains a description of the variant and the light conditions

Source Fig3. cw-ESR and DEER of iLOV-Sort. Col. A-E contains the cwESR data of free GGGGC-SL peptide and the iLOV-Sort measured at room temperature. Col. G-L contains the DEER time trace and distance distribution for iLOV-Sort after light activation measured at 60K.

Source Fig4. DEER time traces and distance distributions for dCRY WT and L405E/C416N variants. Col. A-E and G-K contains information regarding the L405E/C416N variant and WT protein respectively.

Source Fig5. The DEER distance distributions for the H378 variants of dCRY.

File name: Supplementary Data 2
Description: Source Data for Figure S7 contained in a power point file.

Source Figure S7: Light-induced dCRY binding to Timeless (TIM) and TIM degradation. Slides 2-9, 11-15, and 17-23 contain the raw images from which S7b, S7d, and 7e have been determined. Each gel lane has been labeled to indicate the protein and the tag/label used. Slides 2-9 show all fluorescent gel images for the immunoprecipitation between CRY-WT, sortag, delta variants and Tim. Both lysate samples and pulldown samples are included. Slides 11-15 show the degradation level of TIM. Slides 17-23 show the degradation level of CRY variants.
