# Supplementary material for: Tuning flavin environment to detect and control light-induced conformational switching in Drosophila cryptochrome
Source: Commun Biol. 2021 Feb 26;4:249. doi: 10.1038/s42003-021-01766-2 (PMC7910608; doi:10.1038/s42003-021-01766-2)
Supplement: Supplementary file 5 — Supplementary Data 2 [file 42003_2021_1766_MOESM5_ESM.pptx]

## Slide 1
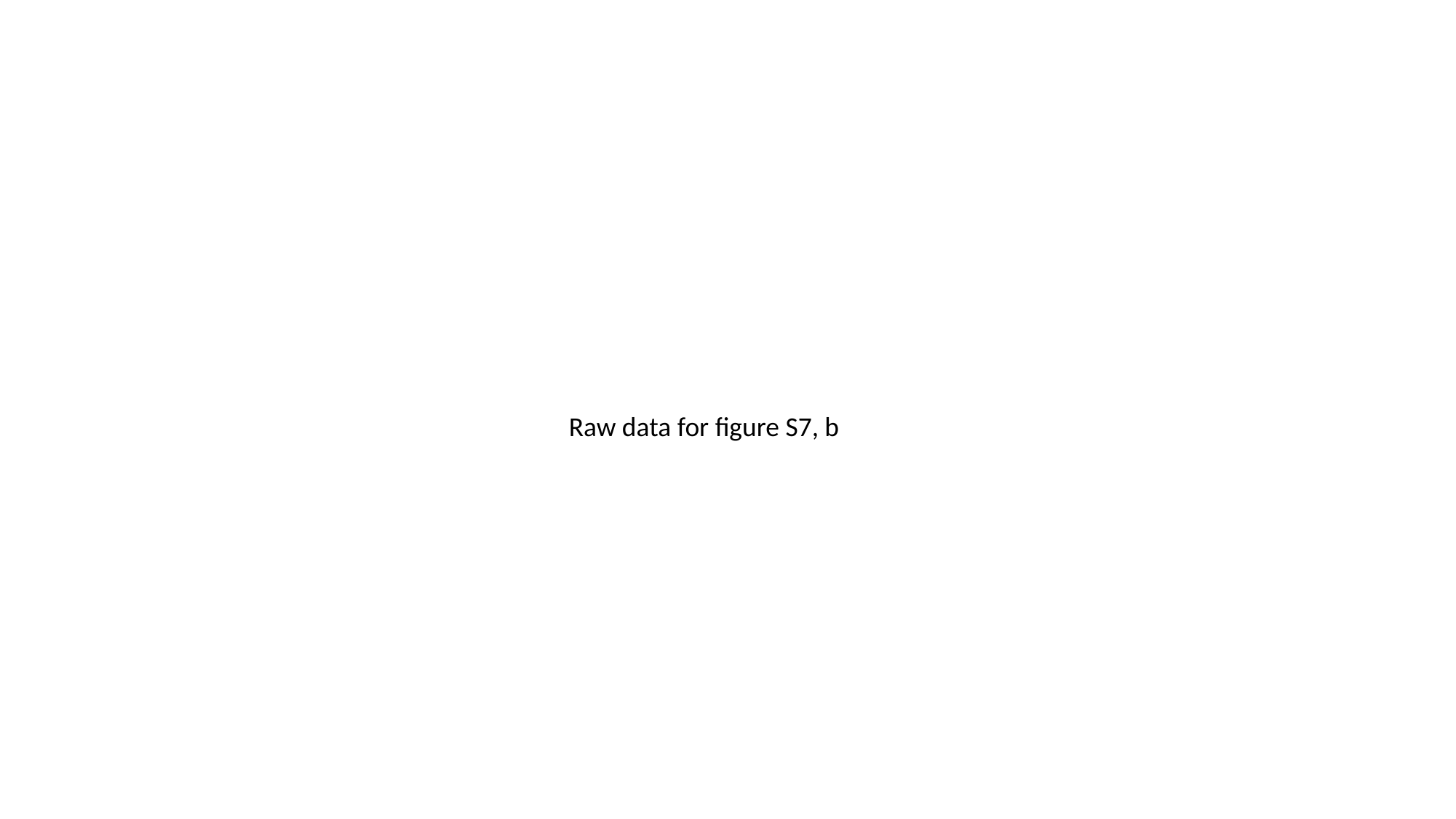

Raw data for figure S7, b

## Slide 2
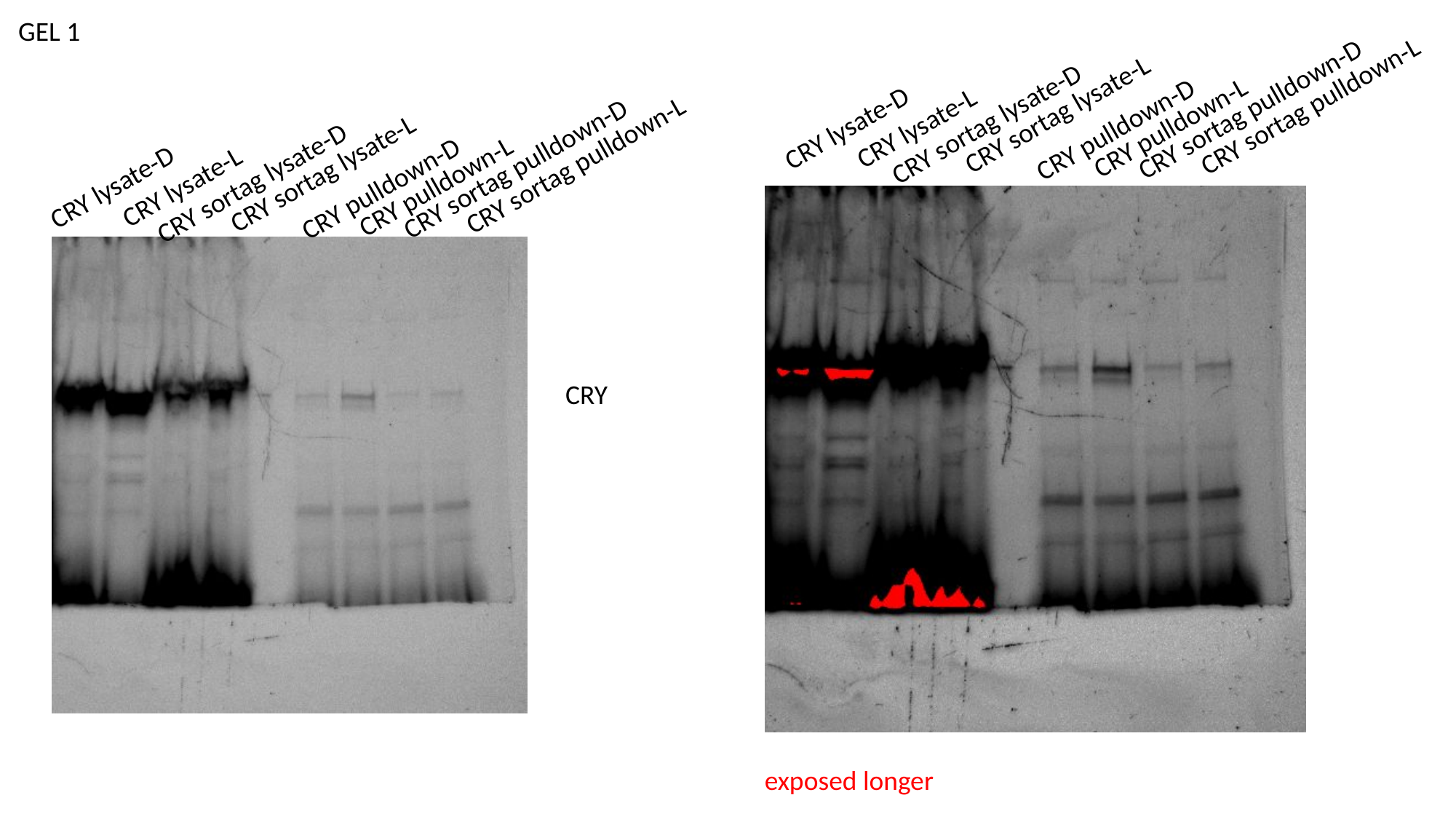

GEL 1
CRY sortag pulldown-L
CRY sortag pulldown-D
CRY sortag lysate-L
CRY sortag lysate-D
CRY pulldown-L
CRY lysate-D
CRY lysate-L
CRY pulldown-D
CRY sortag pulldown-L
CRY sortag pulldown-D
CRY sortag lysate-L
CRY sortag lysate-D
CRY pulldown-L
CRY lysate-D
CRY lysate-L
CRY pulldown-D
CRY
exposed longer

## Slide 3
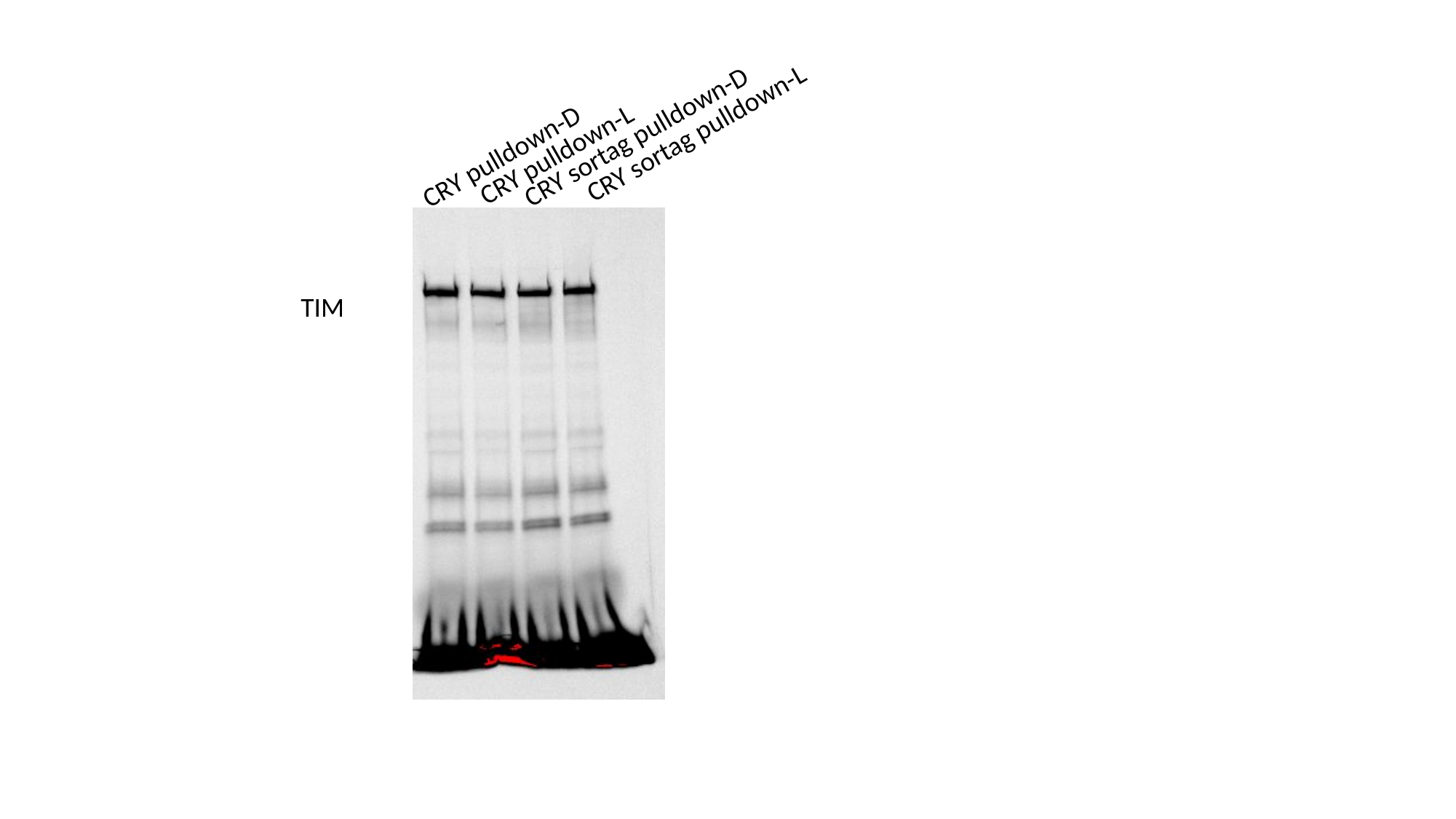

CRY sortag pulldown-L
CRY sortag pulldown-D
CRY pulldown-L
CRY pulldown-D
TIM

## Slide 4
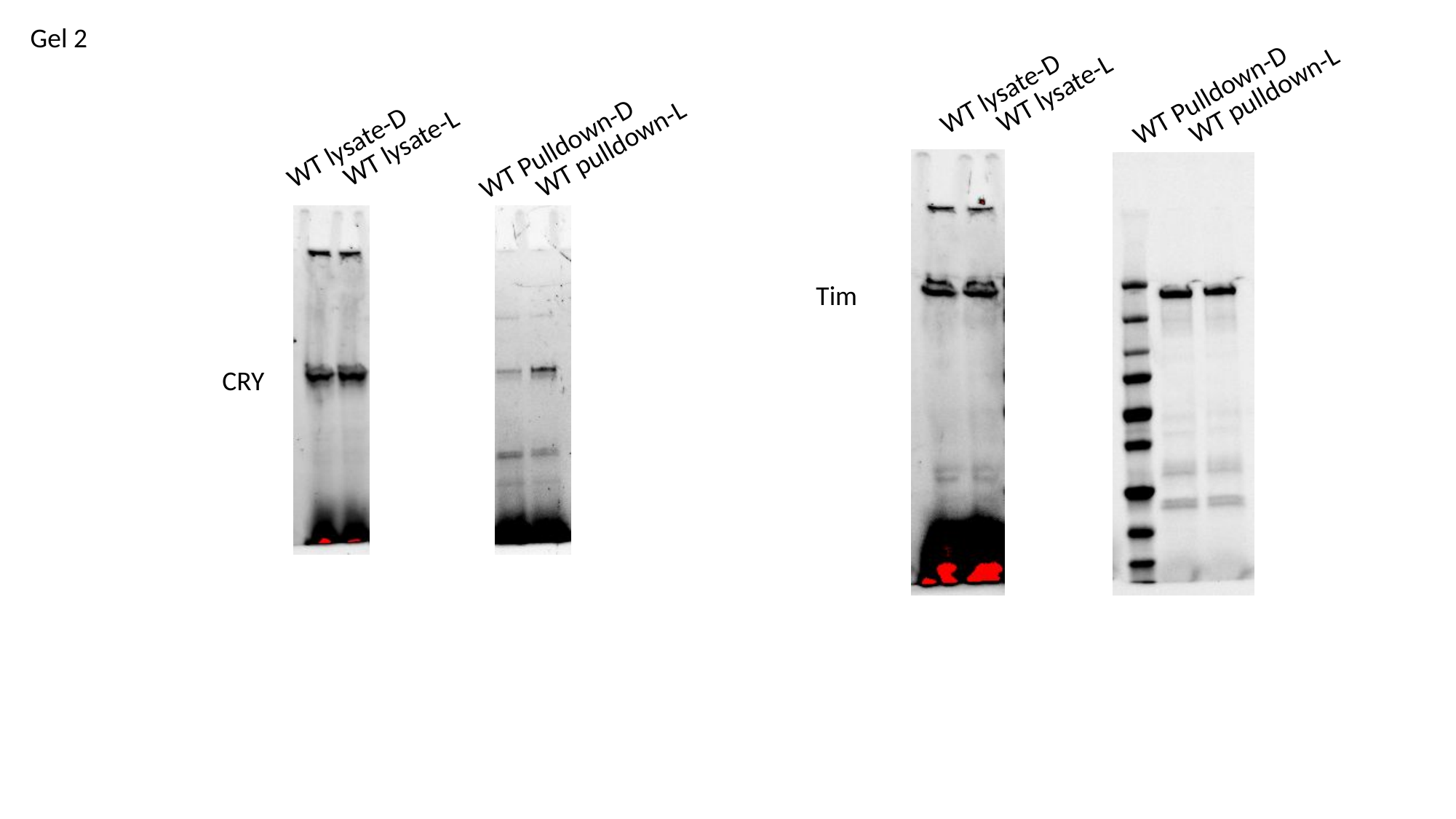

Gel 2
WT lysate-D
WT lysate-L
WT Pulldown-D
WT pulldown-L
Tim
WT lysate-D
WT lysate-L
WT Pulldown-D
WT pulldown-L
CRY

## Slide 5
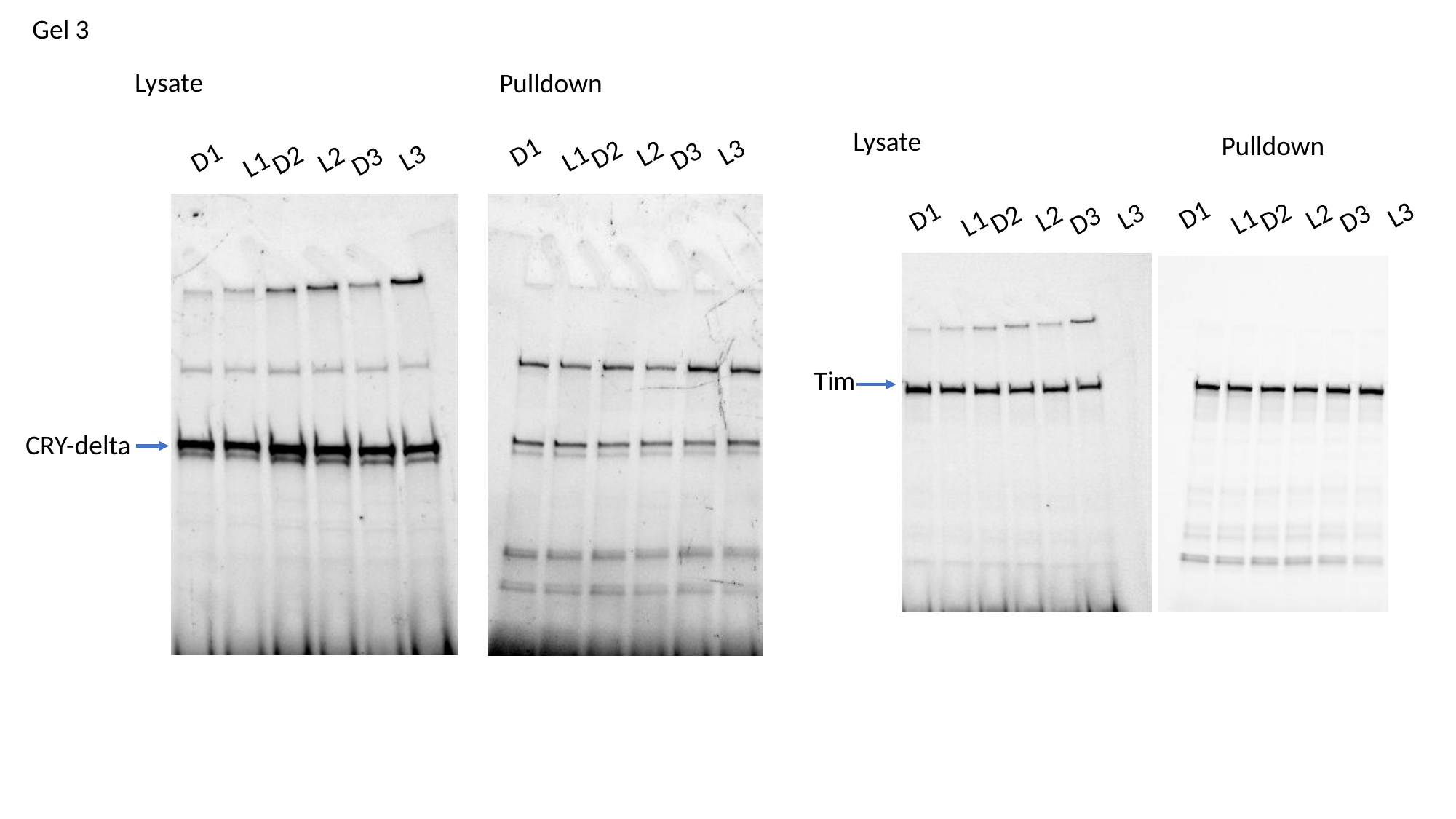

Gel 3
Lysate
Pulldown
Lysate
D1
L3
L2
D2
D3
L1
Tim
Pulldown
D1
L3
L2
D2
D3
L1
D1
L3
L2
D2
D3
D1
L3
L1
L2
D2
D3
L1
CRY-delta

## Slide 6
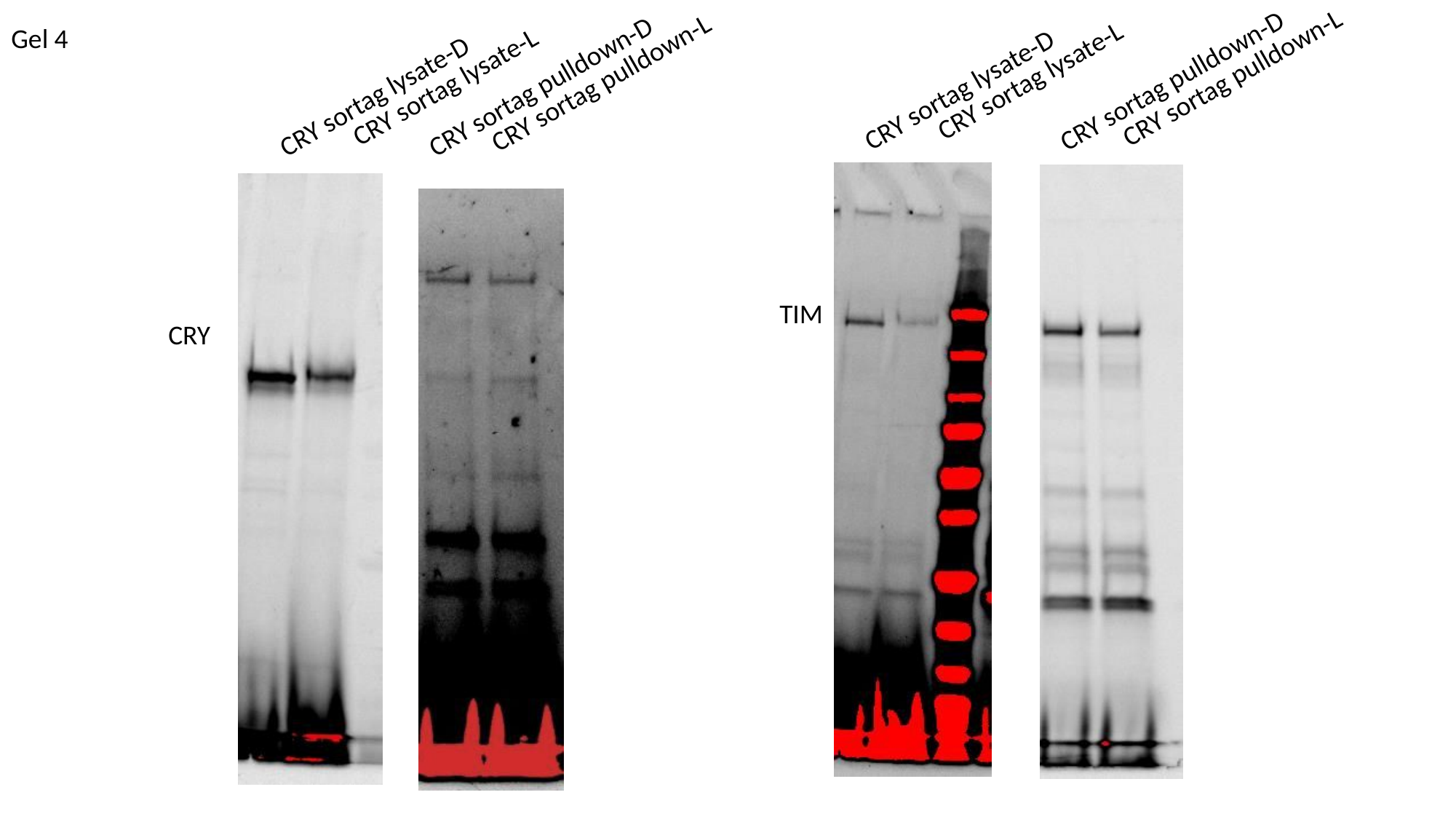

Gel 4
CRY sortag pulldown-L
CRY sortag lysate-L
CRY sortag pulldown-D
CRY sortag lysate-D
TIM
CRY sortag pulldown-L
CRY sortag pulldown-D
CRY sortag lysate-L
CRY sortag lysate-D
CRY

## Slide 7
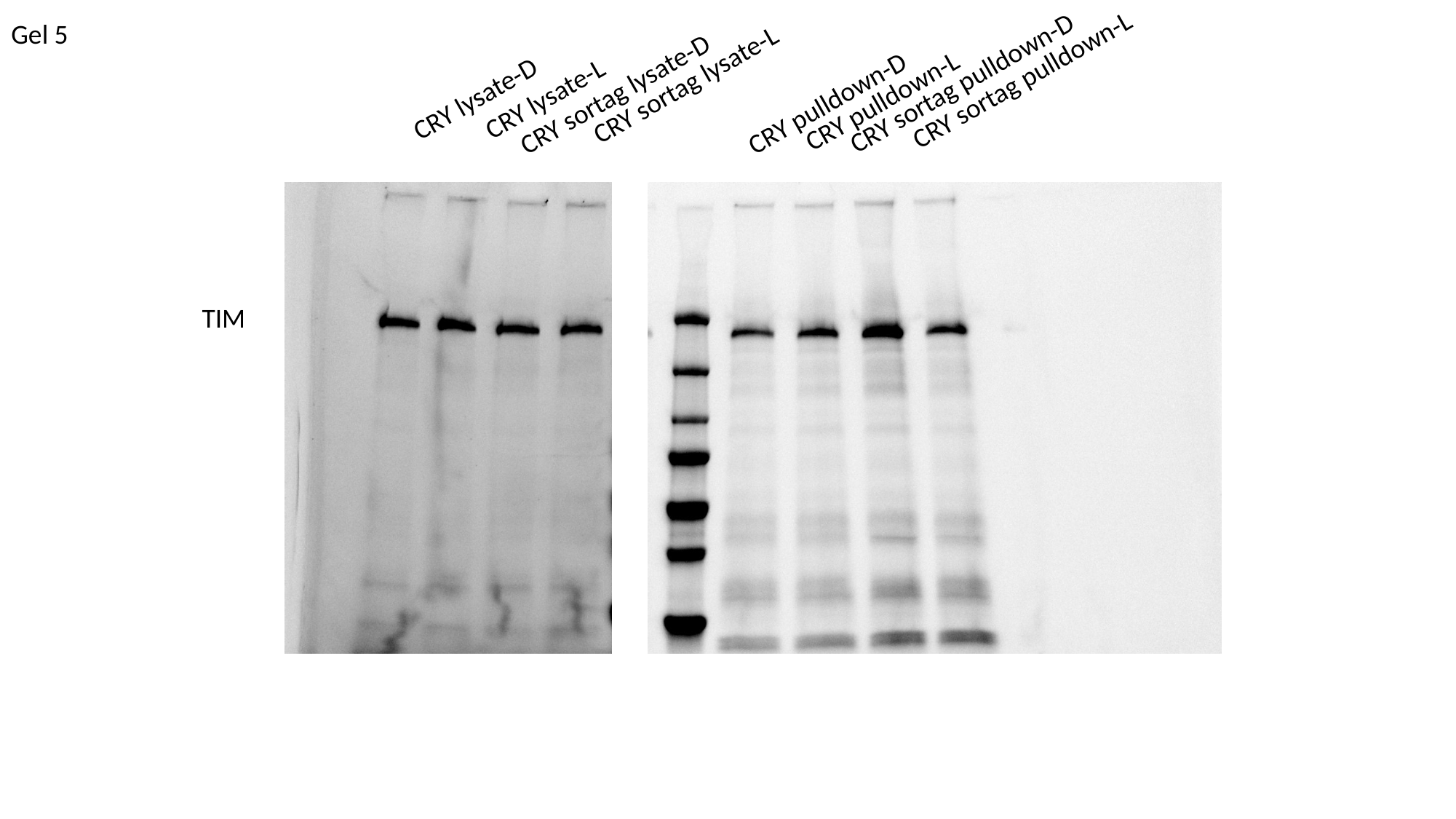

Gel 5
CRY sortag pulldown-L
CRY sortag pulldown-D
CRY sortag lysate-L
CRY sortag lysate-D
CRY lysate-D
CRY lysate-L
CRY pulldown-L
CRY pulldown-D
TIM

## Slide 8
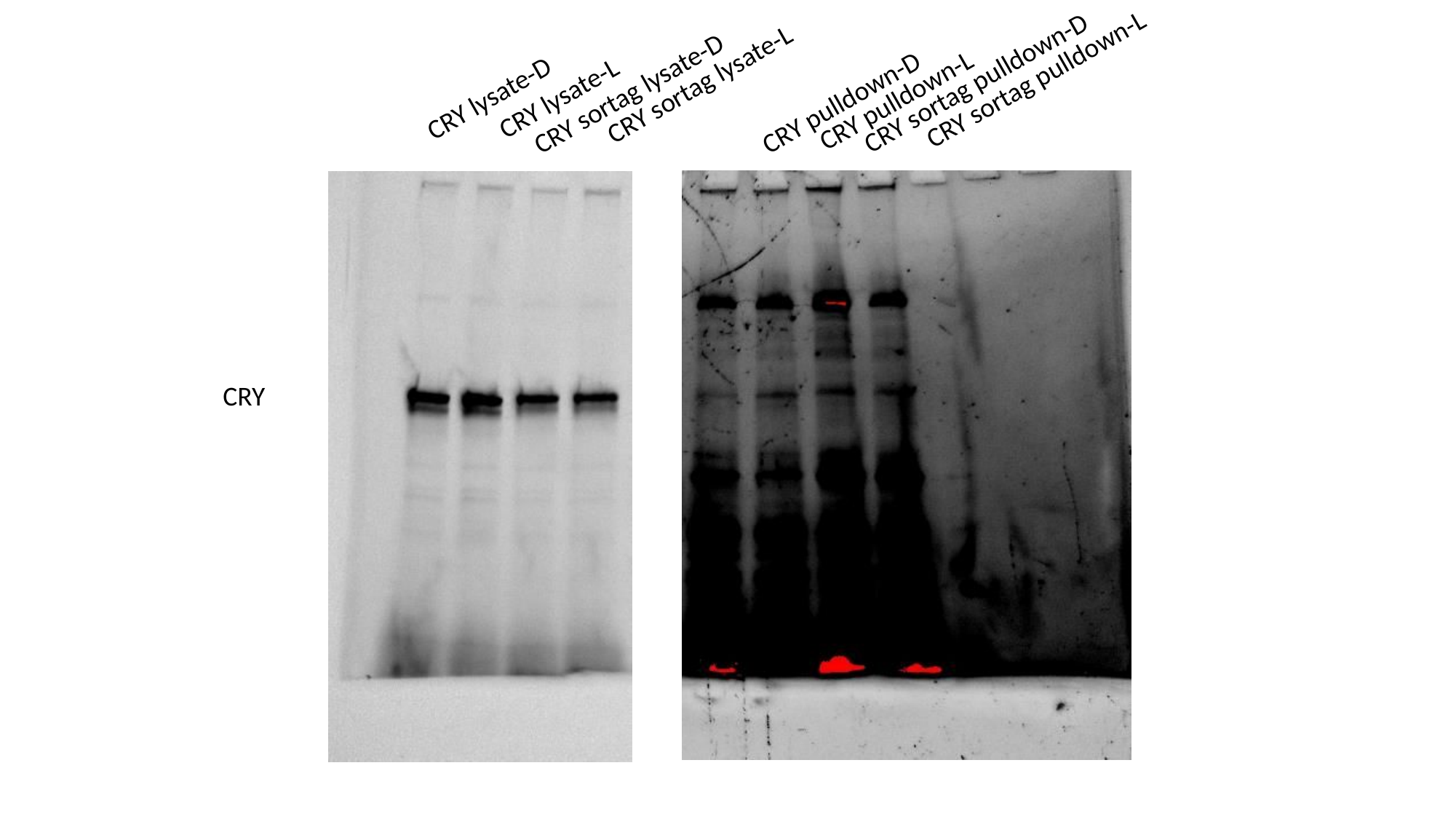

CRY sortag pulldown-L
CRY sortag pulldown-D
CRY sortag lysate-L
CRY sortag lysate-D
CRY lysate-D
CRY lysate-L
CRY pulldown-L
CRY pulldown-D
CRY

## Slide 9
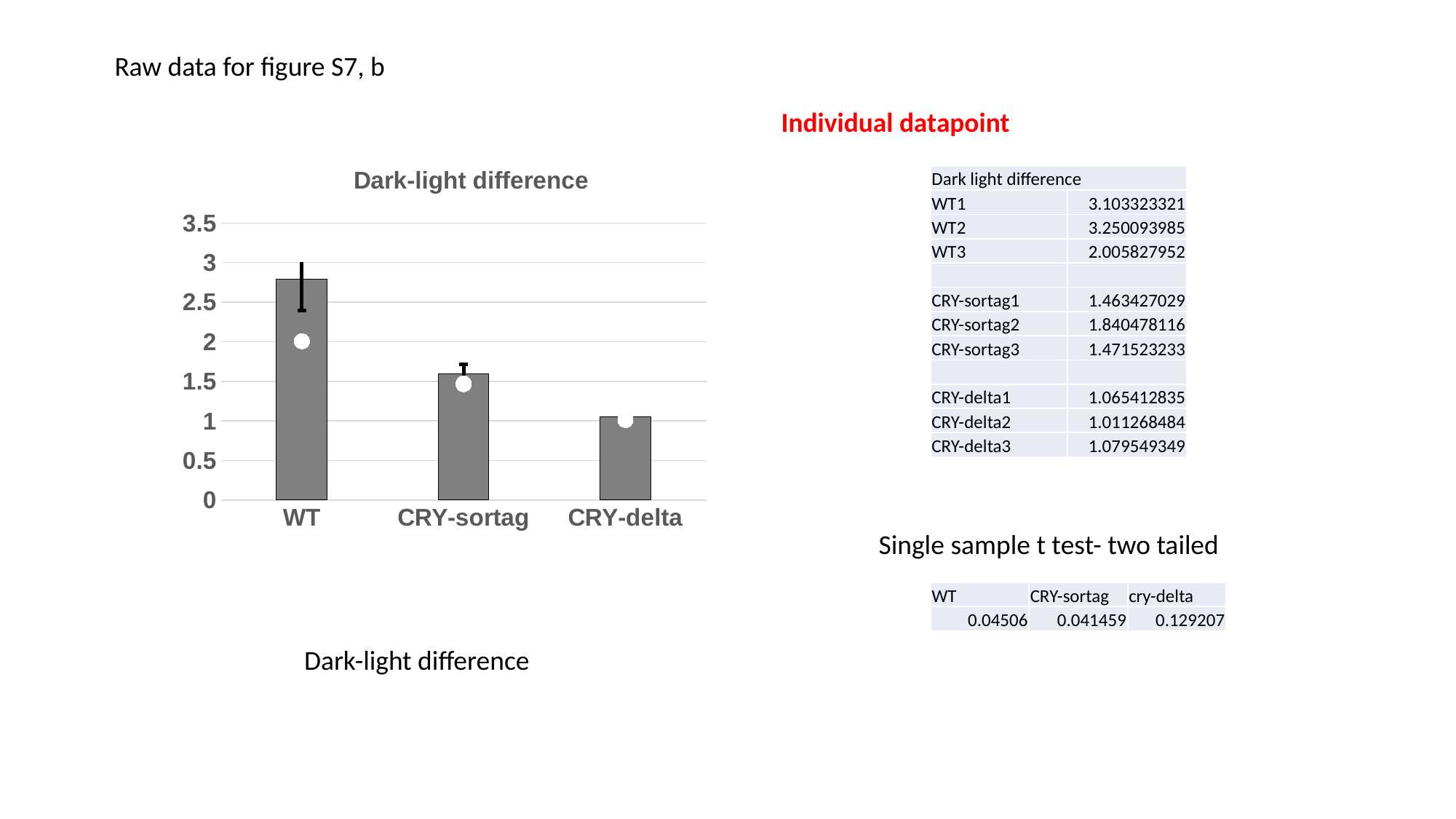

Raw data for figure S7, b
Individual datapoint
### Chart: Dark-light difference
| Category | Dark-light difference | | | |
|---|---|---|---|---|
| WT | 2.7864150861918016 | 3.103323321367197 | 3.2500939847255212 | 2.005827952482687 |
| CRY-sortag | 1.5918094595564938 | 1.4634270292056732 | 1.8404781164132527 | 1.471523233050555 |
| CRY-delta | 1.052076889337118 | 1.0654128347486662 | 1.0112684840252855 | 1.0795493492374022 || Dark light difference | |
| --- | --- |
| WT1 | 3.103323321 |
| WT2 | 3.250093985 |
| WT3 | 2.005827952 |
| | |
| CRY-sortag1 | 1.463427029 |
| CRY-sortag2 | 1.840478116 |
| CRY-sortag3 | 1.471523233 |
| | |
| CRY-delta1 | 1.065412835 |
| CRY-delta2 | 1.011268484 |
| CRY-delta3 | 1.079549349 |
Single sample t test- two tailed
| WT | CRY-sortag | cry-delta |
| --- | --- | --- |
| 0.04506 | 0.041459 | 0.129207 |

## Slide 10
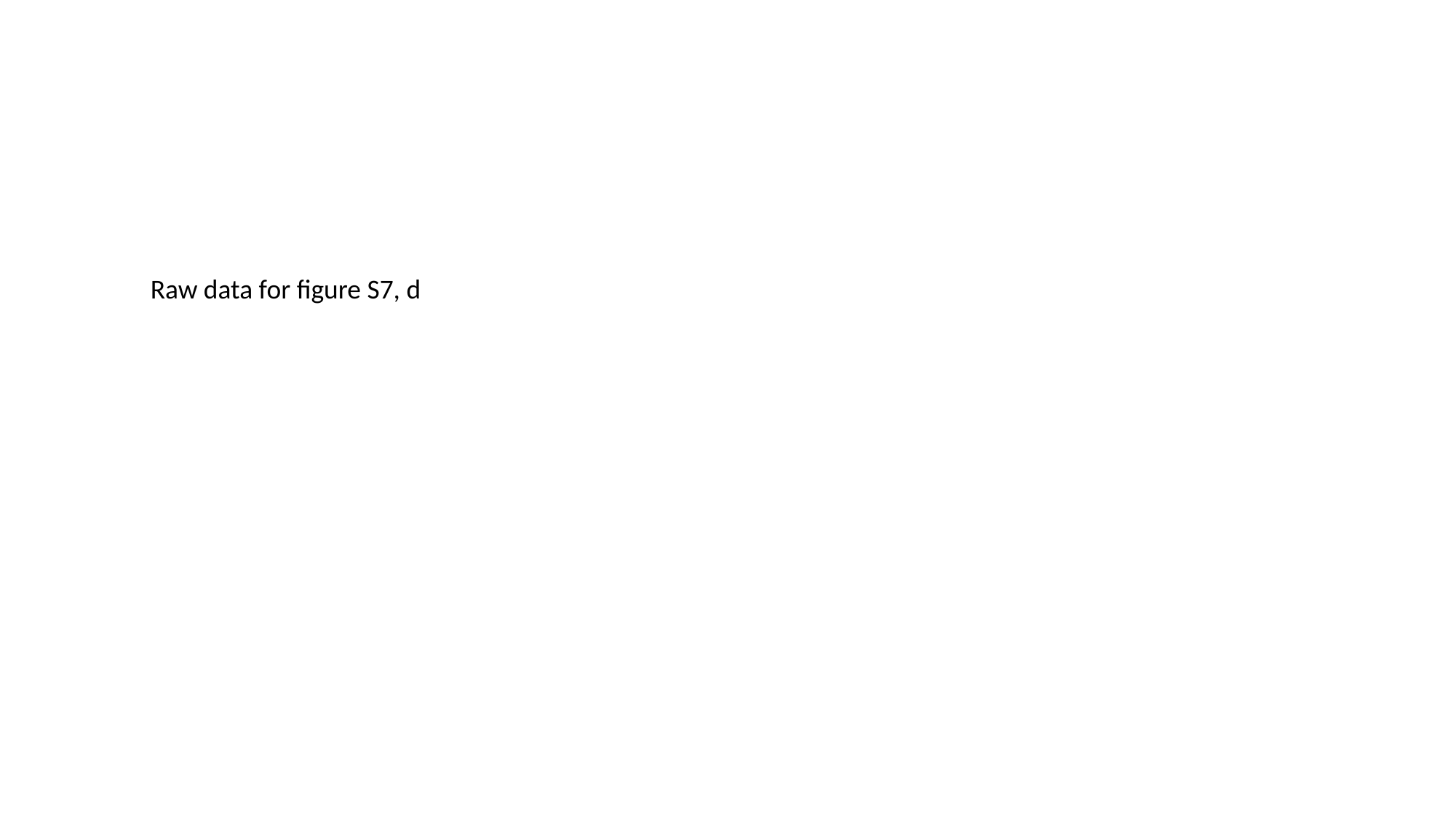

Raw data for figure S7, d

## Slide 11
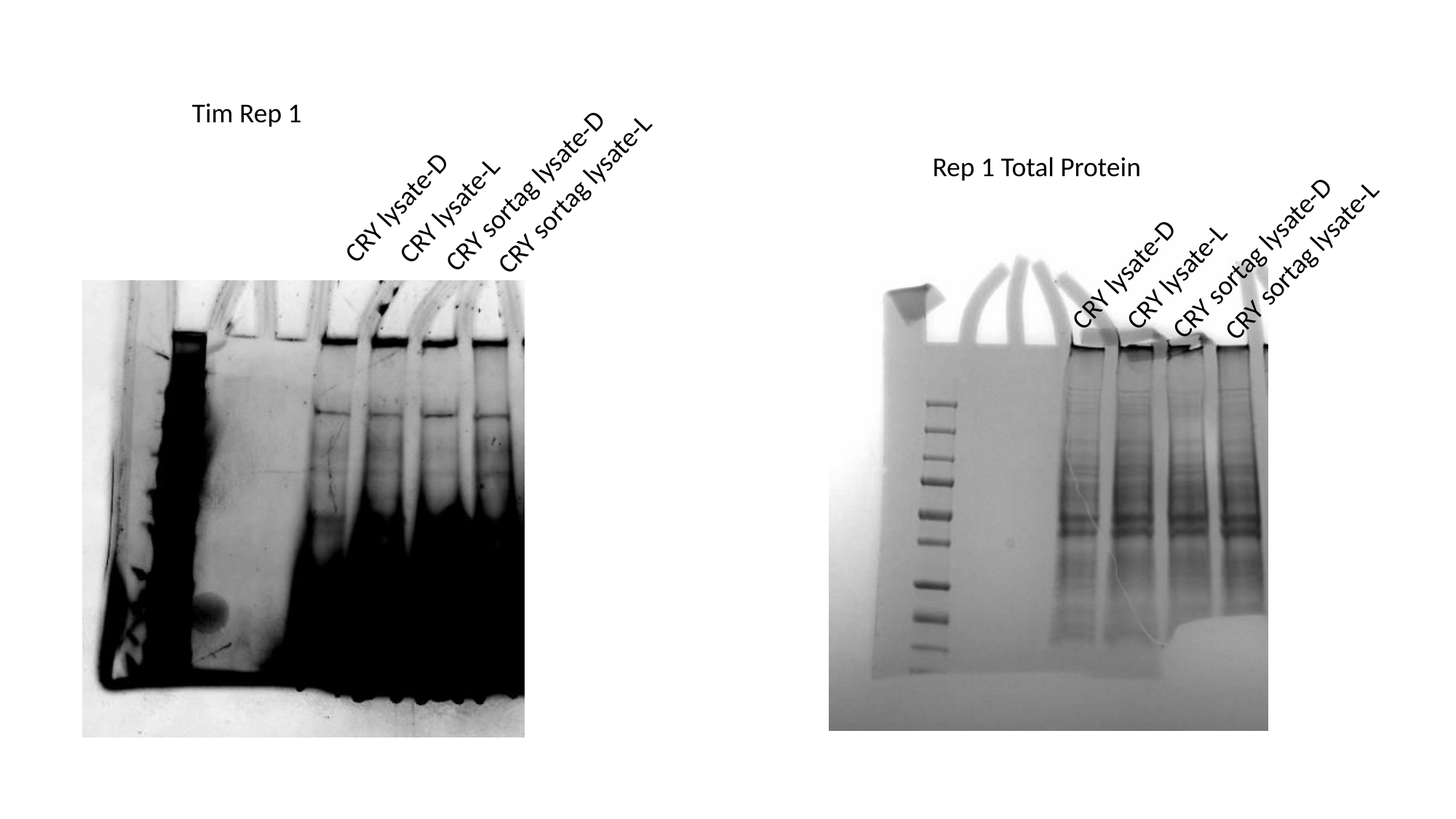

Tim Rep 1
Rep 1 Total Protein
CRY sortag lysate-D
CRY sortag lysate-L
CRY lysate-D
CRY lysate-L
CRY sortag lysate-D
CRY sortag lysate-L
CRY lysate-D
CRY lysate-L

## Slide 12
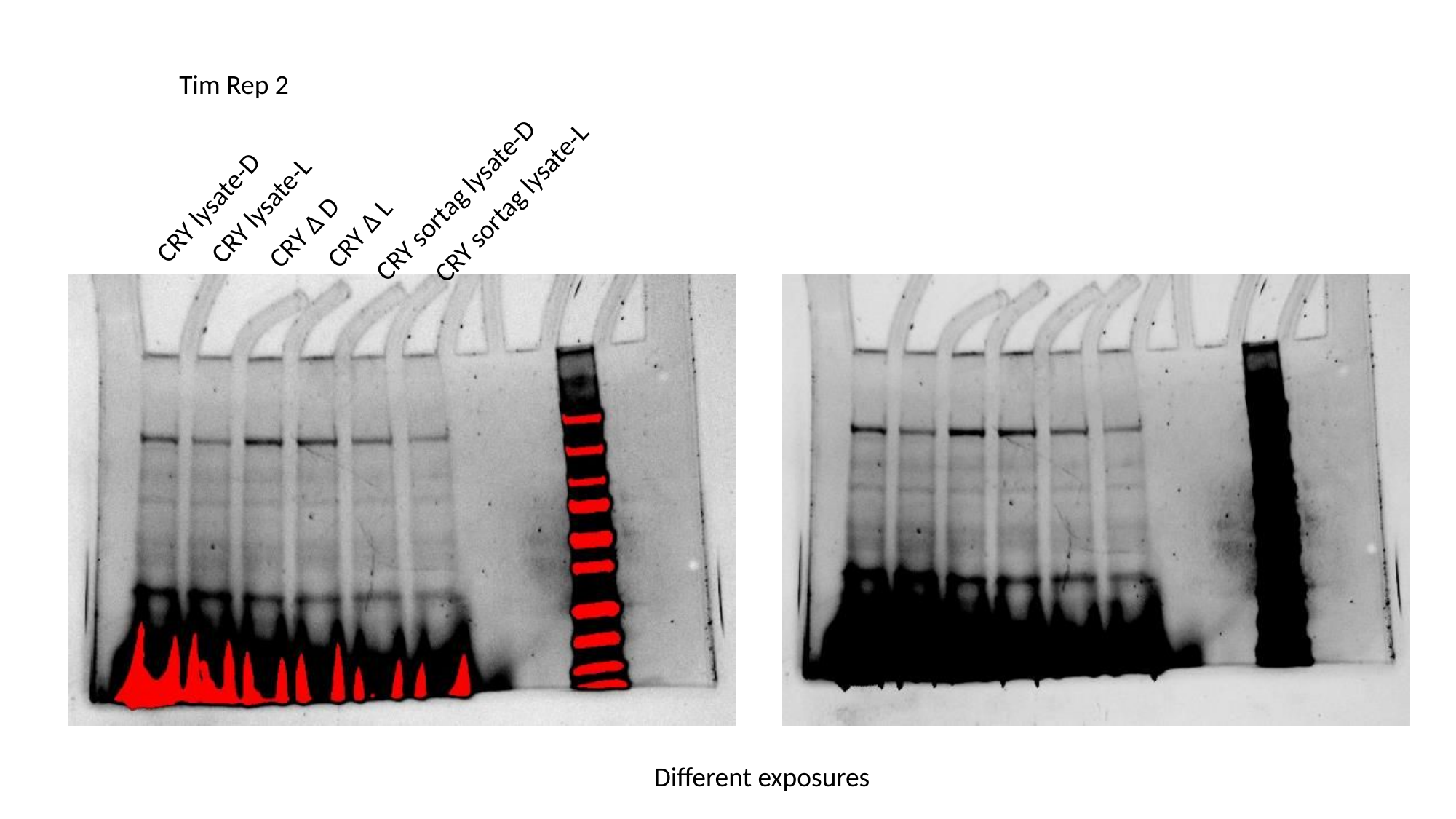

Tim Rep 2
CRY sortag lysate-D
CRY sortag lysate-L
CRY lysate-D
CRY lysate-L
CRY Δ D
CRY Δ L
Different exposures

## Slide 13
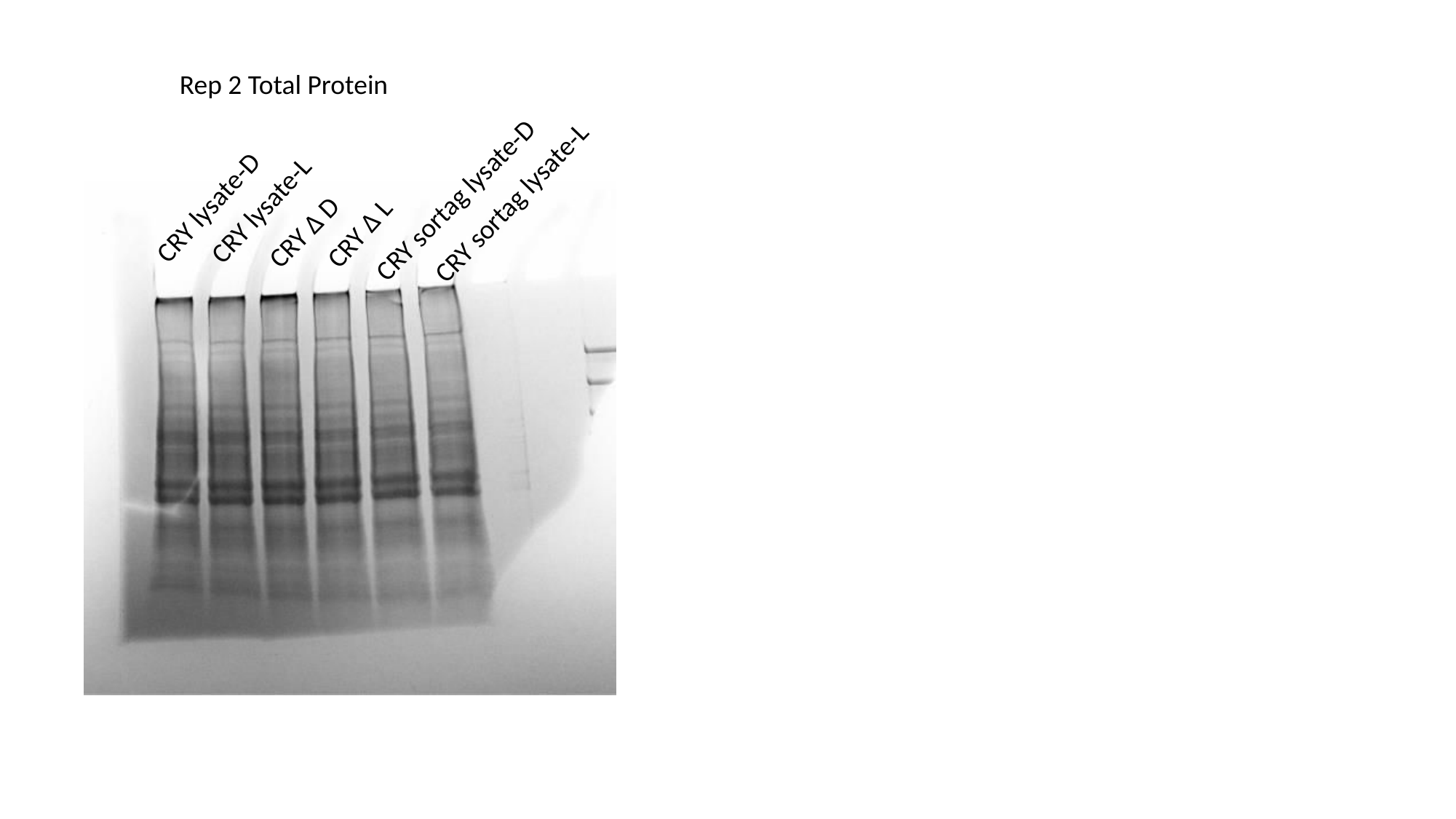

Rep 2 Total Protein
CRY sortag lysate-D
CRY sortag lysate-L
CRY lysate-D
CRY lysate-L
CRY Δ D
CRY Δ L

## Slide 14
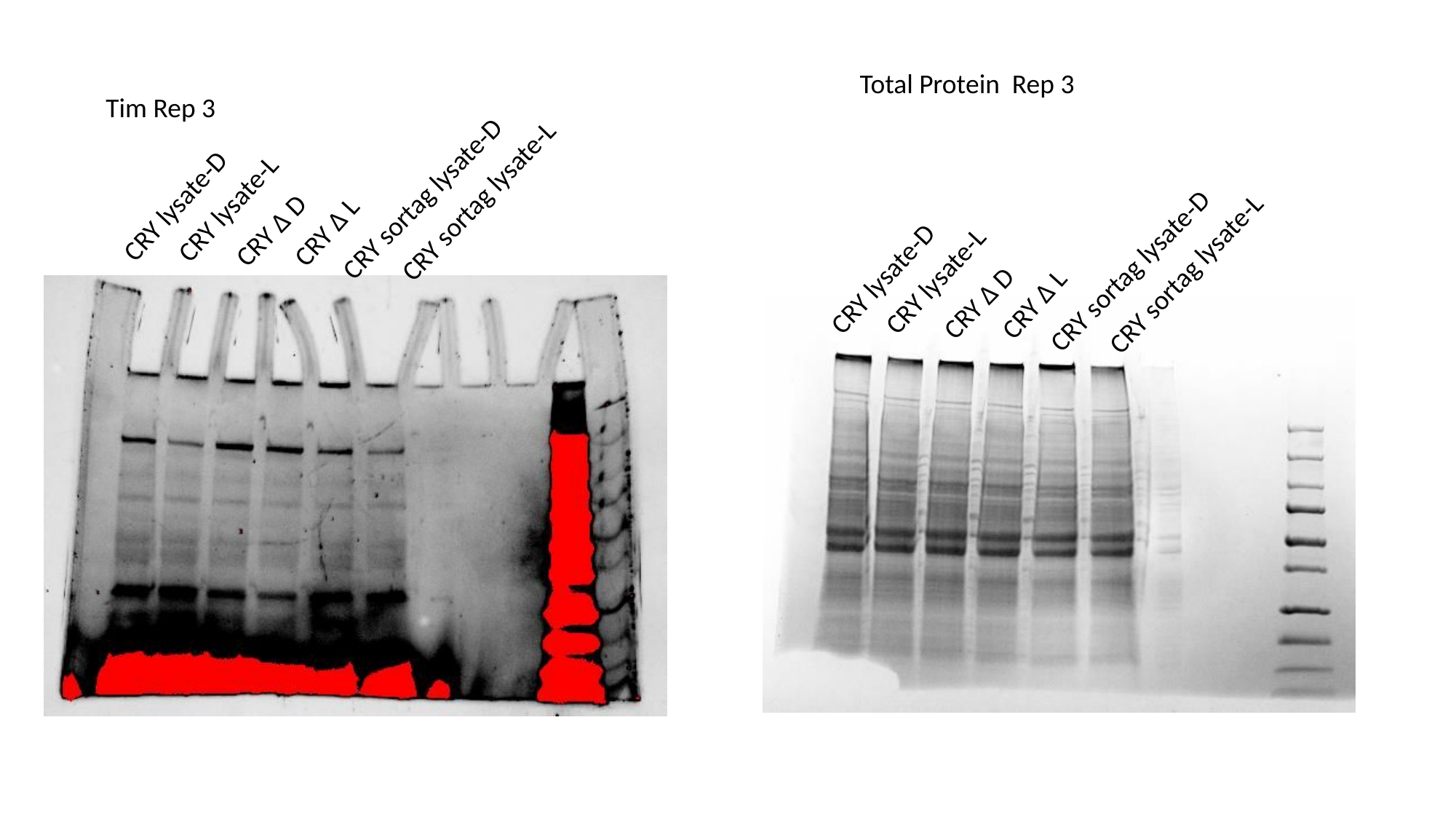

Total Protein Rep 3
Tim Rep 3
CRY sortag lysate-D
CRY sortag lysate-L
CRY lysate-D
CRY lysate-L
CRY Δ D
CRY Δ L
CRY sortag lysate-D
CRY sortag lysate-L
CRY lysate-D
CRY lysate-L
CRY Δ D
CRY Δ L

## Slide 15
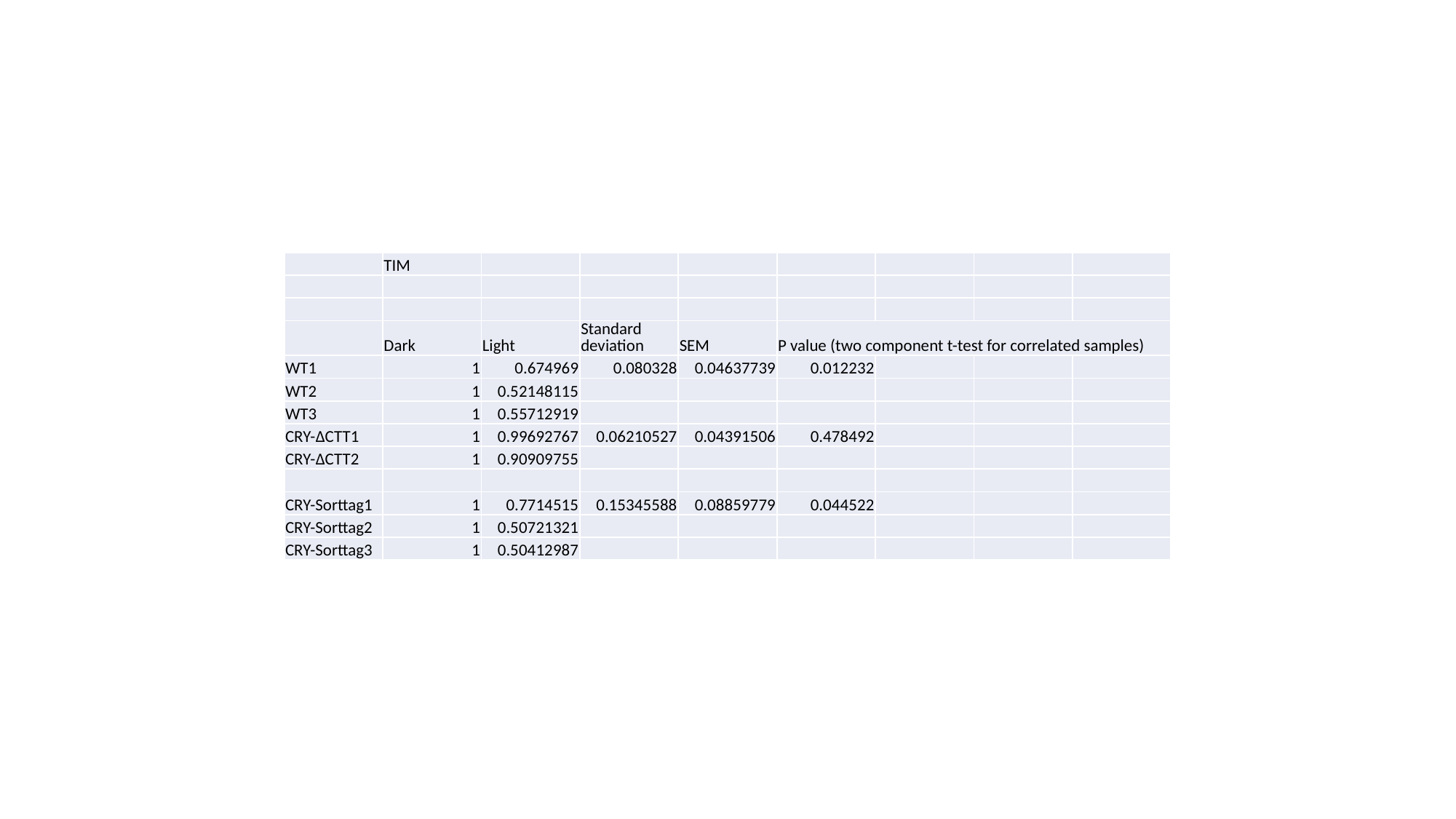

| | TIM | | | | | | | |
| --- | --- | --- | --- | --- | --- | --- | --- | --- |
| | | | | | | | | |
| | | | | | | | | |
| | Dark | Light | Standard deviation | SEM | P value (two component t-test for correlated samples) | | | |
| WT1 | 1 | 0.674969 | 0.080328 | 0.04637739 | 0.012232 | | | |
| WT2 | 1 | 0.52148115 | | | | | | |
| WT3 | 1 | 0.55712919 | | | | | | |
| CRY-ΔCTT1 | 1 | 0.99692767 | 0.06210527 | 0.04391506 | 0.478492 | | | |
| CRY-ΔCTT2 | 1 | 0.90909755 | | | | | | |
| | | | | | | | | |
| CRY-Sorttag1 | 1 | 0.7714515 | 0.15345588 | 0.08859779 | 0.044522 | | | |
| CRY-Sorttag2 | 1 | 0.50721321 | | | | | | |
| CRY-Sorttag3 | 1 | 0.50412987 | | | | | | |

## Slide 16
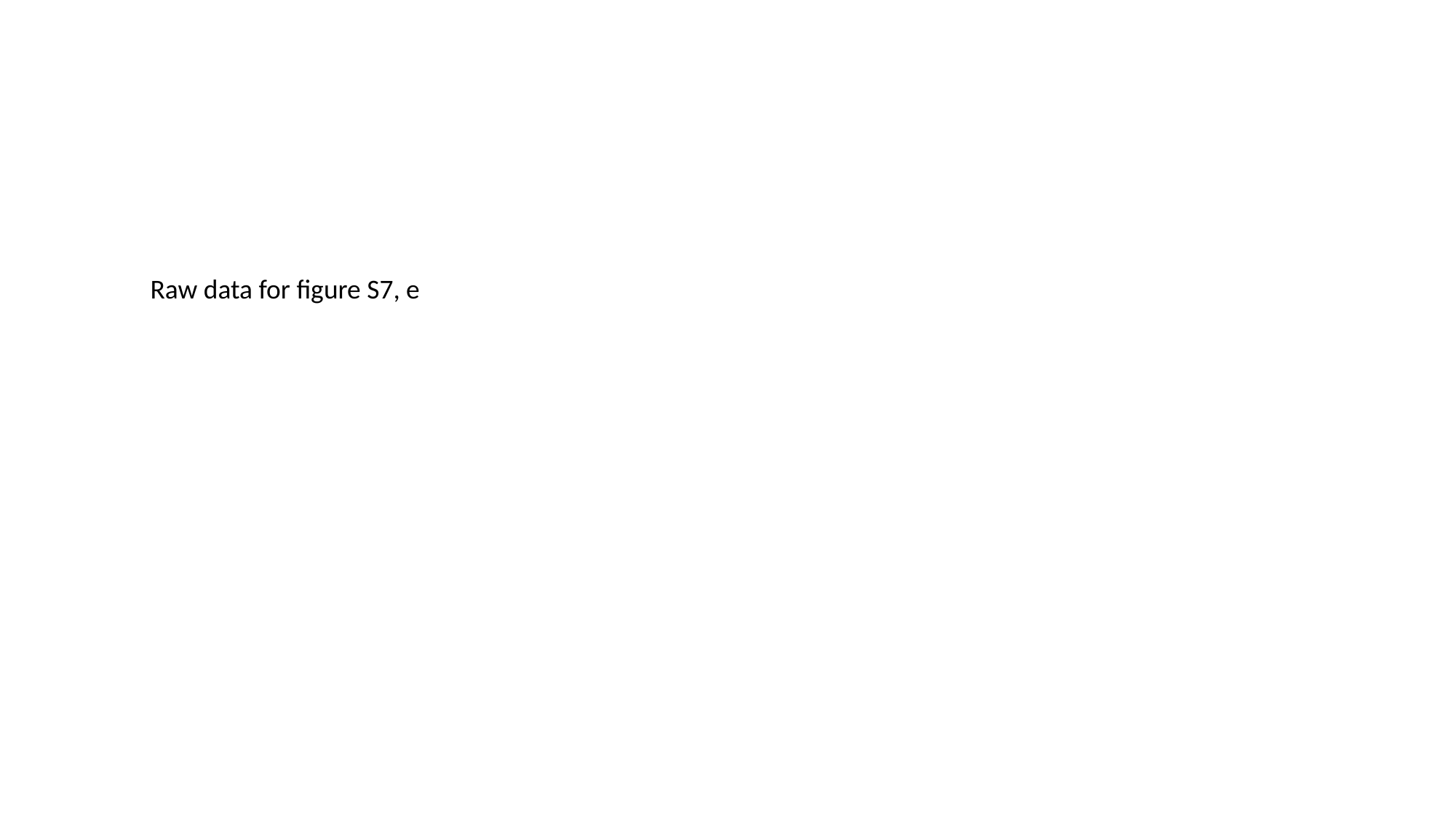

Raw data for figure S7, e

## Slide 17
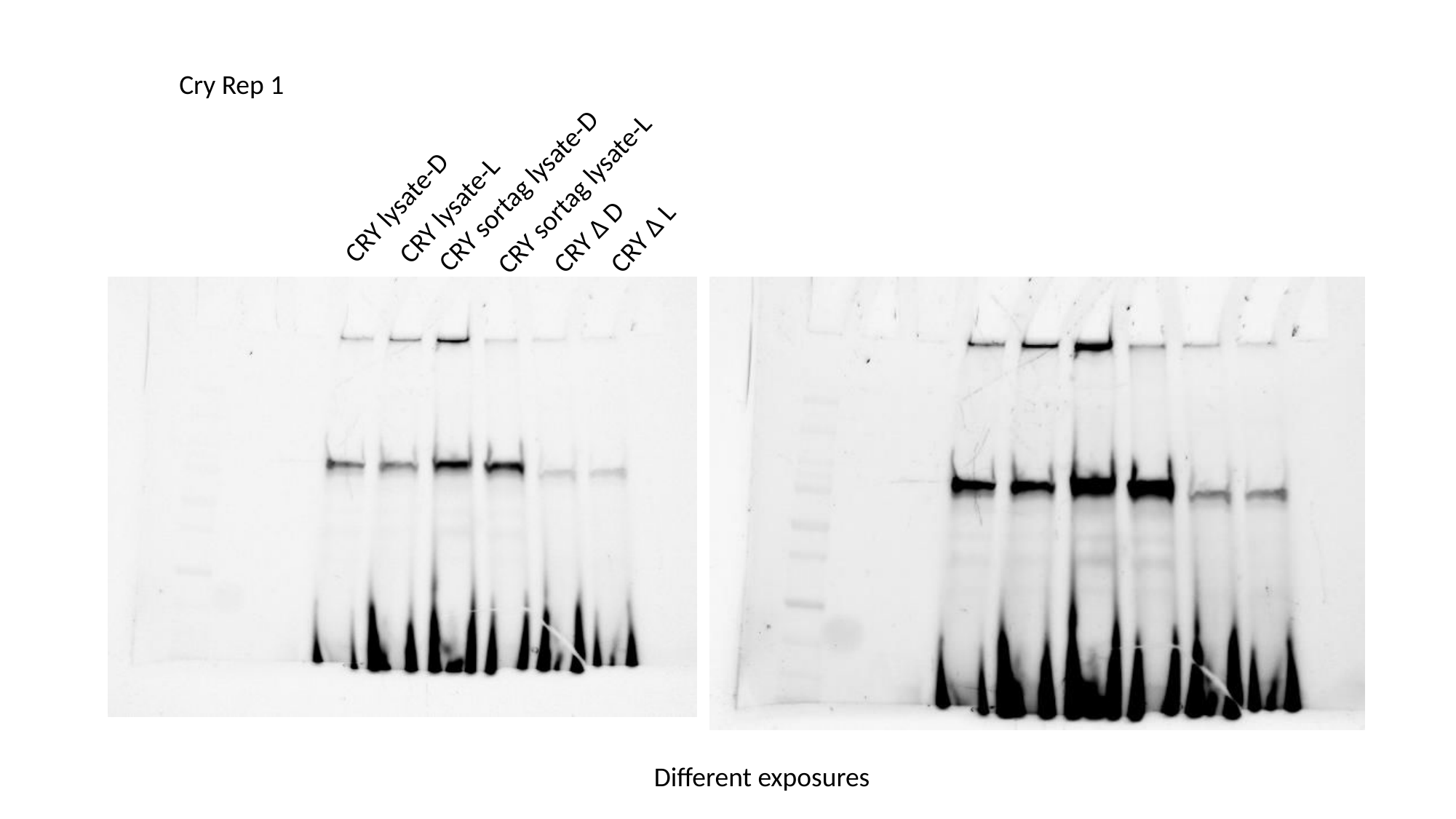

Cry Rep 1
CRY sortag lysate-D
CRY sortag lysate-L
CRY lysate-D
CRY lysate-L
CRY Δ L
CRY Δ D
Different exposures

## Slide 18
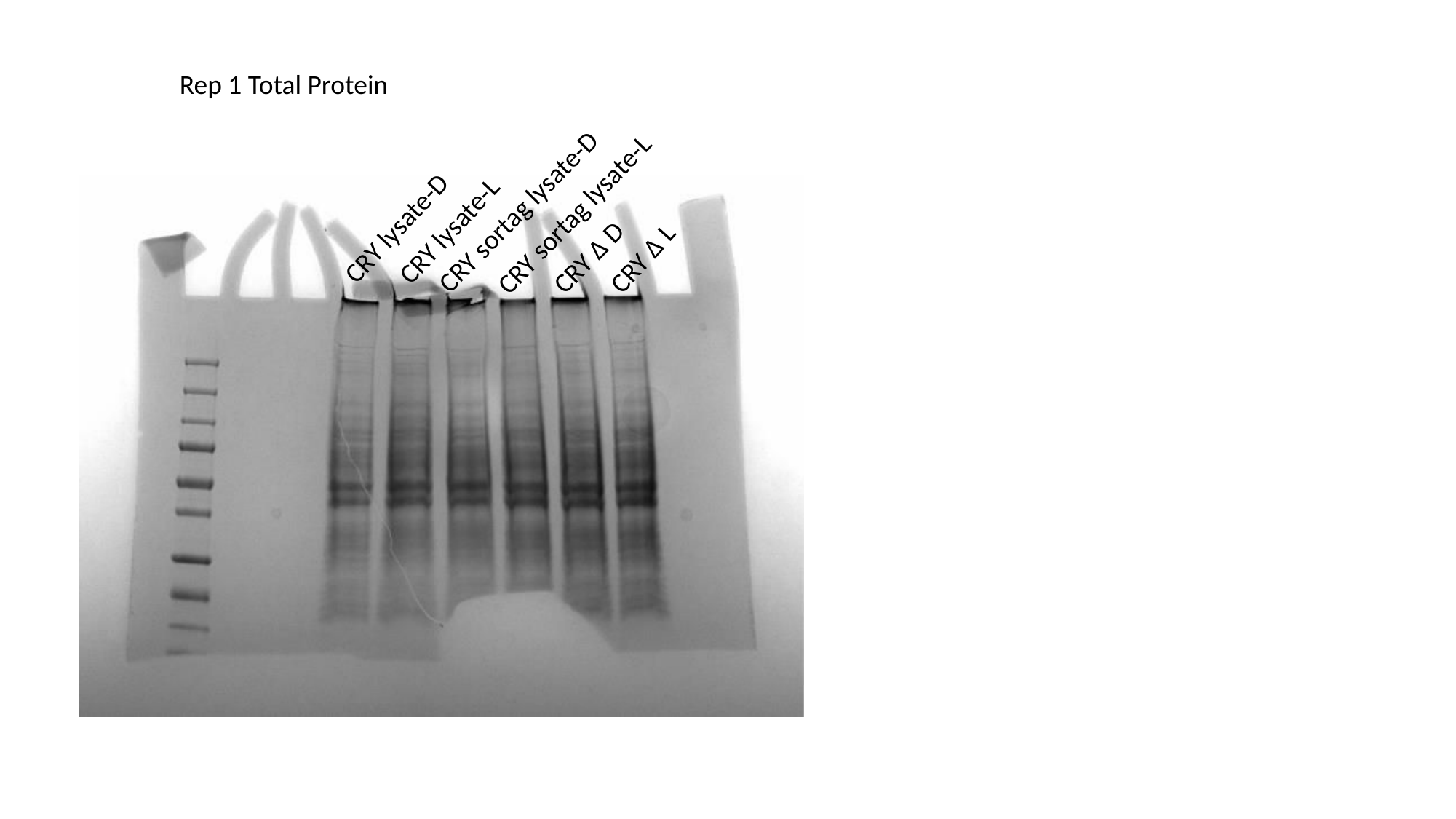

Rep 1 Total Protein
CRY sortag lysate-D
CRY sortag lysate-L
CRY lysate-D
CRY lysate-L
CRY Δ L
CRY Δ D

## Slide 19
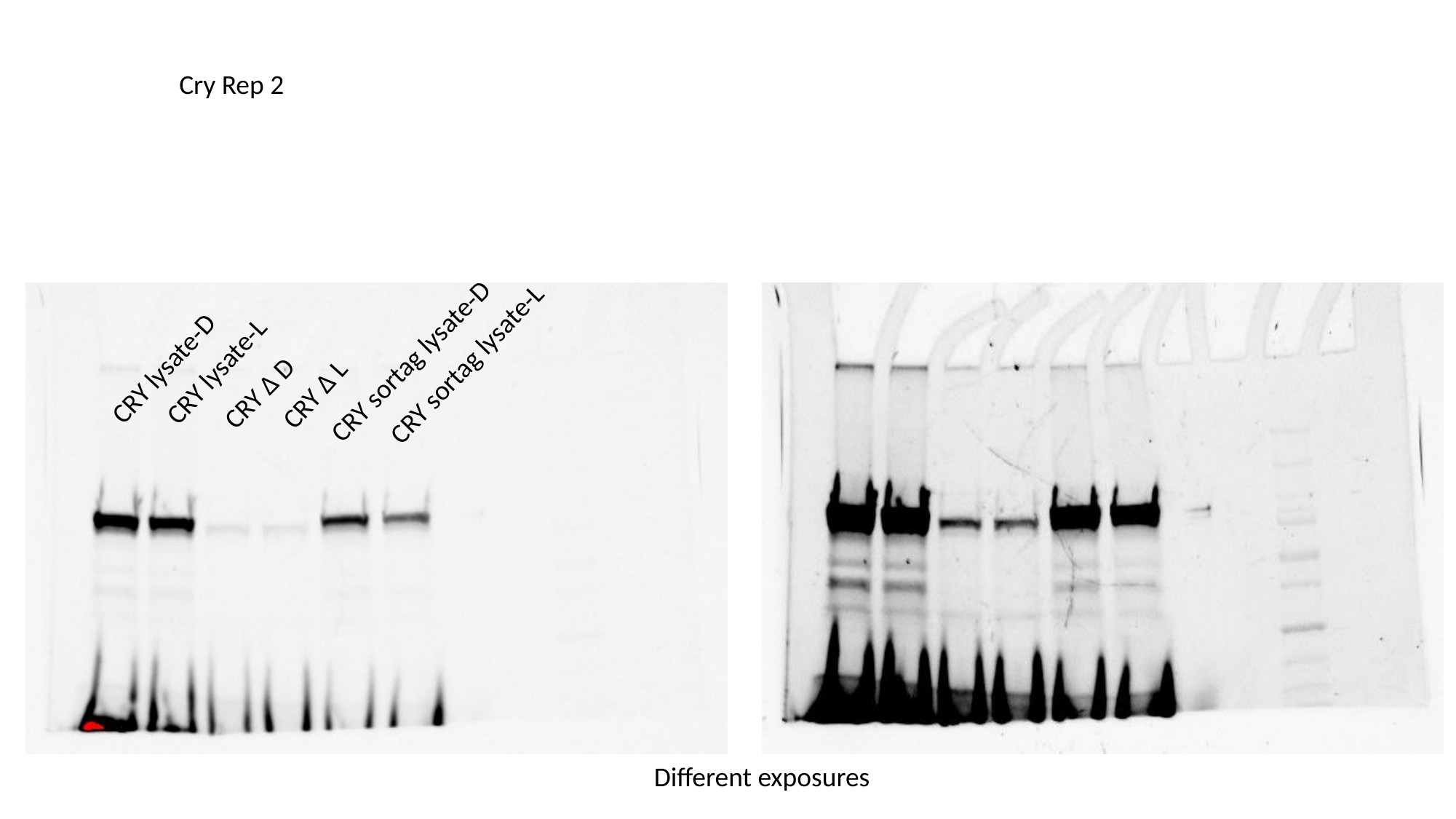

Cry Rep 2
CRY sortag lysate-D
CRY sortag lysate-L
CRY lysate-D
CRY lysate-L
CRY Δ D
CRY Δ L
Different exposures

## Slide 20
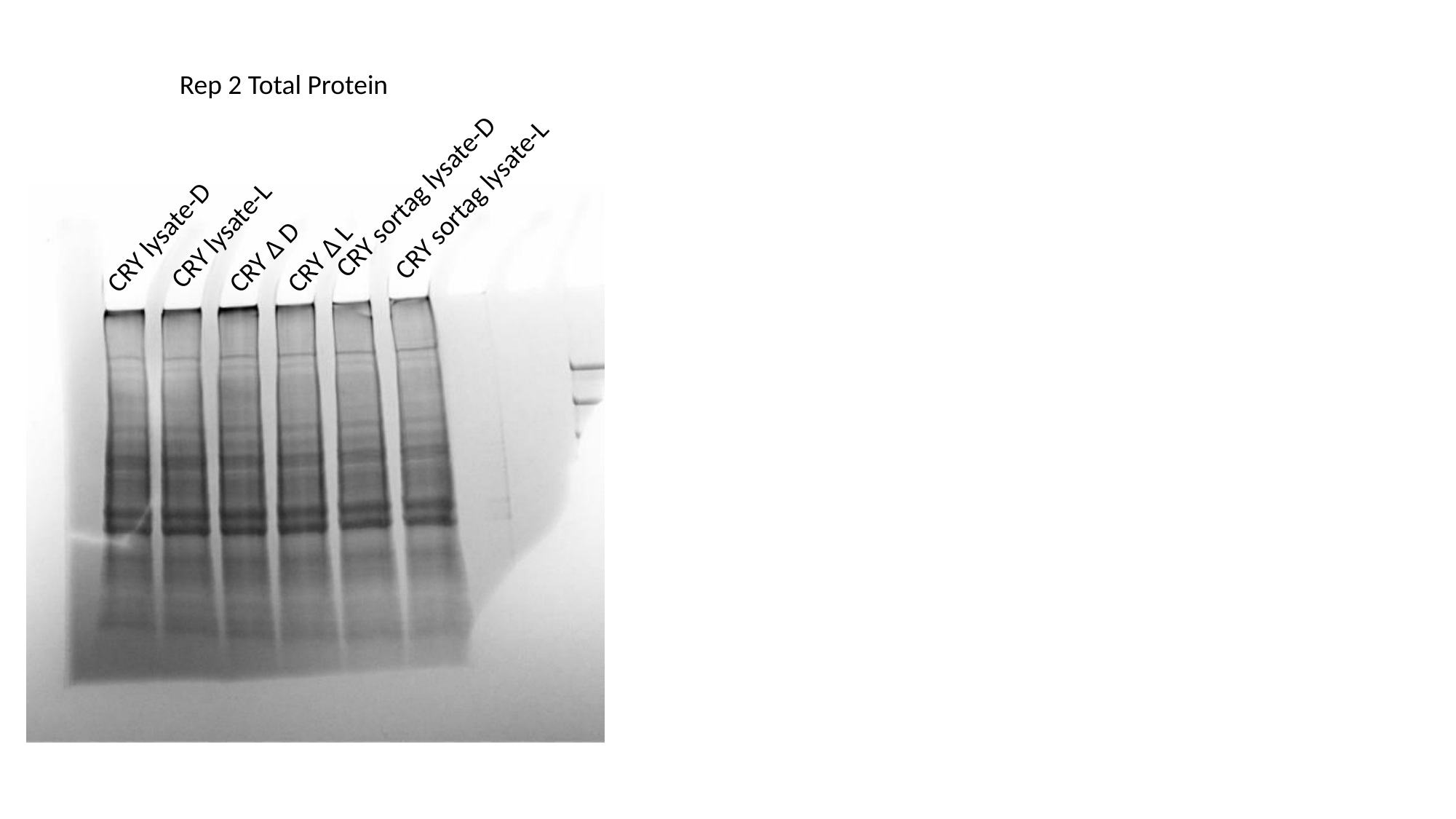

Rep 2 Total Protein
CRY sortag lysate-D
CRY sortag lysate-L
CRY lysate-L
CRY lysate-D
CRY Δ D
CRY Δ L

## Slide 21
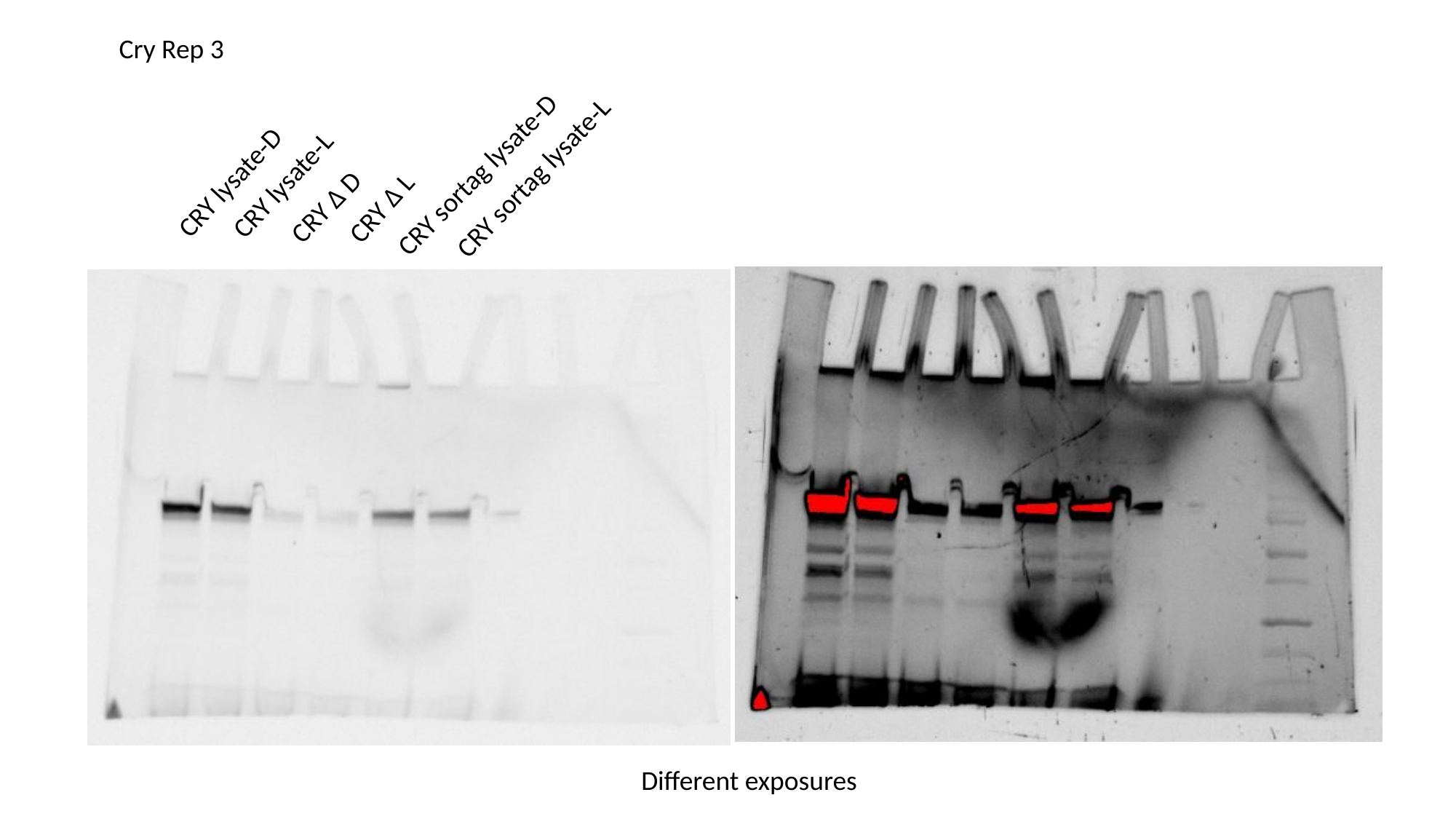

Cry Rep 3
CRY sortag lysate-D
CRY sortag lysate-L
CRY lysate-D
CRY lysate-L
CRY Δ D
CRY Δ L
Different exposures

## Slide 22
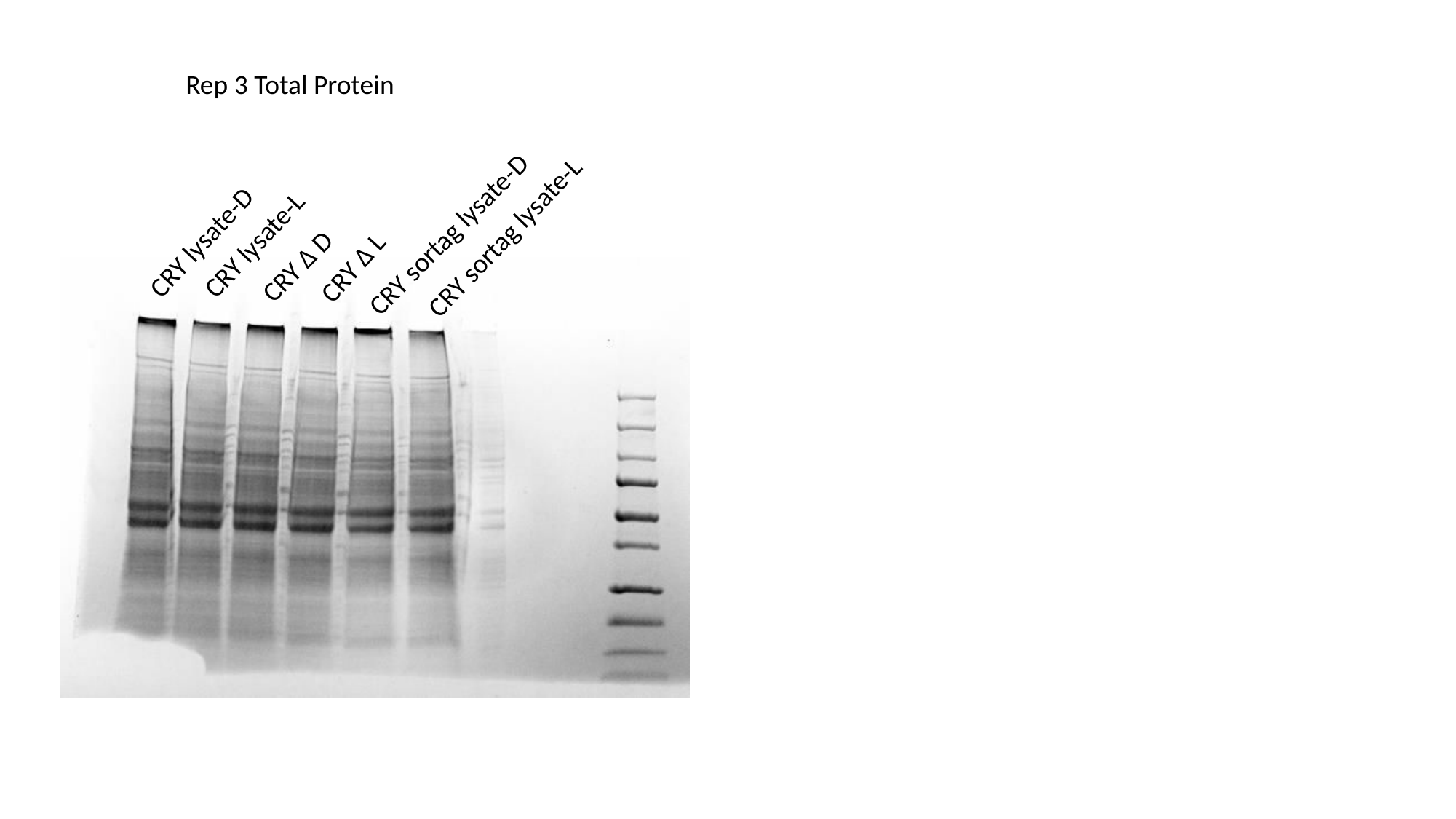

Rep 3 Total Protein
CRY sortag lysate-D
CRY sortag lysate-L
CRY lysate-D
CRY lysate-L
CRY Δ D
CRY Δ L

## Slide 23
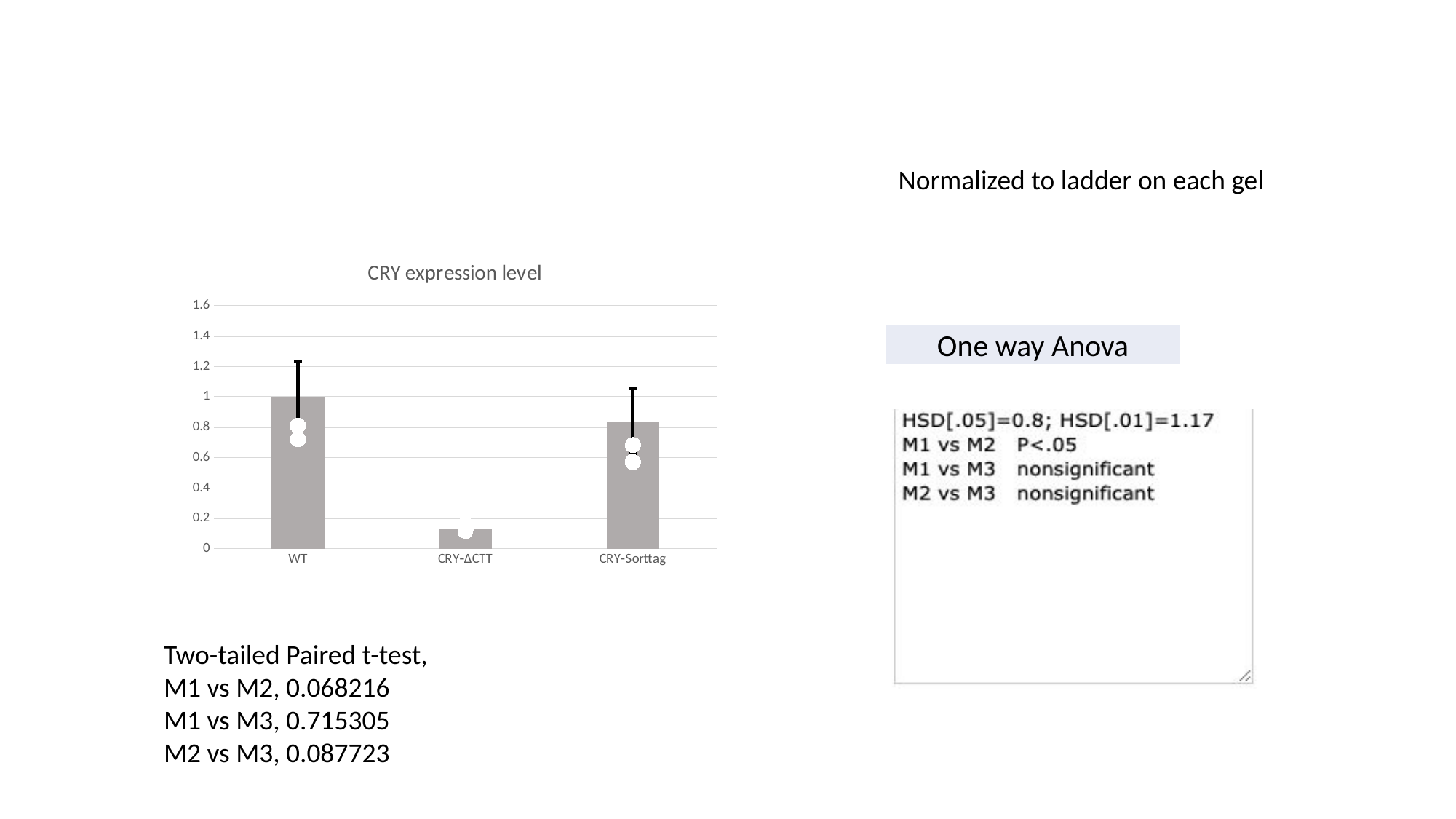

Normalized to ladder on each gel
### Chart: CRY expression level
| Category | Protein level | | | |
|---|---|---|---|---|
| WT | 1.0000000000235891 | 0.7216084807126615 | 0.8101057598392122 | 1.4682857595188936 |
| CRY-ΔCTT | 0.13308529204686015 | 0.11773899510816546 | 0.15716631700516157 | 0.12435056402725339 |
| CRY-Sorttag | 0.839846690096277 | 1.2650744816253279 | 0.5713722929258721 | 0.6830932957376308 || One way Anova |
| --- |
Two-tailed Paired t-test,
M1 vs M2, 0.068216
M1 vs M3, 0.715305
M2 vs M3, 0.087723
