# Supplementary material for: Tuning flavin environment to detect and control light-induced conformational switching in Drosophila cryptochrome
Source: Commun Biol. 2021 Feb 26;4:249. doi: 10.1038/s42003-021-01766-2 (PMC7910608; doi:10.1038/s42003-021-01766-2)
Supplement: Supplementary file 1 — Supplementary Information [file 42003_2021_1766_MOESM1_ESM.pdf]

Parts of this Peer Review File have been redacted as indicated to maintain the confidentiality of unpublished data.

Reviewers' comments:

Reviewer #1 (Remarks to the Author):

Summary
The goal of this study was to unravel the influence of the protonation state of the light induced FAD radical (protonated=NSQ versus anionic=ASQ) in Drosophila cryptochrome (dCry) with respect to the release of the CTT, and to elucidate the influence of the amino acid H378. A number of different variants have been produced and measured via pulsed EPR spectroscopy. The first main goal was to generate a variant that stabilize the NSQ and compare it with the WT (which forms an ASQ). This has been achieved by a L405E/C416N mutant. The variant indeed forms a NSQ, which was briefly analyzed by optical and EPR/ENDOR spectroscopy.
The labeling strategy by using sortaseA to covalently attach a spin label to the C-terminus is elegant and state of the art as it does not require any additional cysteine mutations within the dCRY protein. The subsequently performed DEER measurements are backed-up by a crystal structure of the dark state (dCry+Sortase-spin label), which provides precise information about the distance between the FAD and the SL. DEER analysis using dCry in its signaling state showed that the undocked CTT (=signaling state) has a ~40A distance with a broad distance distribution, whereas the docked CTT (=dark state) has a ~30A distance. The latter distance is almost identical to the one obtained from the crystal structure.
Additional DEER experiments using three different H378 variants showed a different behavior of the three variants H378K/N/R with respect to the variants with H378. Whereas H378+ASQ shows CTT displacement, H378 variants seem to stabilize the CTT.

Conclusion:
Studying signal transduction processes of insect cryptochromes is a highly relevant issue and would merit the requirements for publication in "Nature Communications Biology". The finding that only the ASQ but not the NSQ redox state promotes CTT conformational changes is an important novel finding and could help to fully understand molecular details of signal propagation from the FAD chromophore to the surface of the cryptochromes protein. However, the influence of H378 still remains mainly unresolved, but its obvious that it has an important influence. In sum, I would recommend publication after major revision.

Specific comments:

1) ENDOR analysis
- Figure2 legend: Please exchange: “The dCRY L405E/C416N variant has -peaks-“ with “hyperfine couplings” or with “shows frequencies of X MHz around the Proton Larmor frequency”.
- Table1 legend: The weak signal intensity of the H5 hyperfine coupling is mainly because of its large anisotropy. Moreover, the peak at 25.3MHz corresponds to one principal component of the hyperfine tensor. This should be clarified.
- the ENDOR data convincingly show that the FAD radical is protonated in the L405E/C416N variant. A discussion of the different proton hyperfine couplings, perhaps even with respect to other cryptochromes/photolyase data is missing.

2) iLOV construct.
The DEER time trace hardly shows any modulation; therefore, the resulting distance distribution may contain substantial errors (see also the general comment about the analysis of the DEER traces). In addition, most LOV domains show significant (unspecific) dimerization at higher concentrations in vitro, which would result in a number of distances or a broader distance distribution. A control

experiment containing the iLOV/FMN radical alone could clarify this issue.
As a more general question: Why is this sample included in this study as the crystal structure of dCry+Sortase-spin label already showed that the sortase reaction is successful? What is the purpose of this sample?

3) Deer analysis
- Most samples contain a significant amount of "free" spin label (sharp signals in Supporting Fig.10 and 11). I guess that these spins add significantly to the DEER background signal and may obscure any wanted modulations. Perhaps, a second desalting step directly before light irradiation would be helpful.
ELDOR time traces (Figure 4 and S8): Unfortunately, I think that the DEER analysis of the H378 variants is slightly overstated and should be reevaluated carefully. A few examples for this statement:
(i) the DEER analysis of WT and L405E/C416N only show one distance. The analysis of H378R (Fig. 5) however results in only 80% open state (like the WT) although the experimental datasets are almost identical to the one obtained from the WT (Fig. S8). I would recommend to analyze the WT (with FADox), which should give no distance distribution but a background signal, and the WT (FAD ASQ) using the same model to evaluate the quality of the fits.
(ii) The datasets of the three H378K/L405E/C416N and H378R are very similar (Fig. S8), the percentage of open/closed state are quite different (Fig. 5).
(iii) The signal/noise ratio of sample H378N/L405E/C416N is lower than in the other samples. Nevertheless, the error of the fit is considered to be the lowest of all samples (Table2).
(iv) no simulated spectra are presented. It is however quite crucial to show (i) the spectra after baseline subtraction and (ii) the subsequent processing of the spectra. It would be particularly important to overlay the experimental spectra and their respective simulations so that the quality of the fit can be inspected by the reader.
Perhaps, repeating the analysis with a different program without any model (e.g., DEER analysis or other) would help to strengthen the robustness of the fits. Another possibility would be to attenuate the absolute values of the two distance distributions (Table 2) and only provide approximate values as the main statement (H378 variants stabilize the CTT even in the signaling state) remains the same anyway.

4) Interpretation
- H378 variants: I think that another possibility has to be discussed: Triple variants as used in this study are likely to modify the surrounding between FAD and the CTT and could lead to unwanted conformational changes at the CTT and/or could modify signal propagation from the FAD to the CTT. Although this is an unavoidable problem when working with variants, it should at least be stated.
- While the sortase reaction is elegant and has significant advantages (as pointed out in the text), it has also a downside as only the signaling state can be investigated. In this case, the amount of CTT release in the H378 variants under dark-state conditions remain unclear. This should be mentioned somewhere in the Discussion section.

5) Minor comments (without specific order)
- are the samples prepared under aerobic or anaerobic conditions?
- line 474: please remove brackets
- Figure 2 legend: Please exchange H8 with H8alpha
- The alignment and the abbreviations of the variants shown in Fig. 5 and Fig. S8 are very different, which does not support its readability and inhibit direct a comparison. Please adjust.
- line 335: The sentence is misleading as all presented data are recorded in the signaling state.

Reviewer #2 (Remarks to the Author):

This manuscript provides for the first time a complete sequence of events that lead from light-triggered redox changes at the flavin pocket in Dcry to unfolding of the C-terminal domain that triggers biological activation. To do so, the authors have used an elegant, technically flawless spin labelling protocol that specifically attaches a single nitroxide spin probe cleanly at the C-terminal end of the dCRY protein. As a reference spin probe, these authors used the flavin radical (ASQ) for the light – induced dCRY conformation. What is particularly impressive is their characterisation and use of an engineered dCRY variant producing the neutral SQ radical as a dark - state proxy, as this NSQ radical does not allow undocking of the C-terminal domain in response to light and thereby is not biologically active. Pulse dipolar electron-spin resonance spectroscopy allowed identification of significant conformational change and increased motion of the C-terminal domain as a consequence of illumination in the ASQ but not NSQ state, consistent with biological activity. Furthermore these authors performed elegant studies of C-terminal conformational change on a series of mutations at the His378 position, which prior studies had extensively documented in terms of biological activity and impact on flavin redox chemistry. Since all of the results obtained from this study are internally consistent, concur with molecular dynamic simulations, and correspond to or are corroborated by prior studies of biological activation by this receptor, the paper is timely and will be warmly welcomed in a field that has been characterized by much past confusion due to conflicting methodology and differing, largely indirect approaches.

There are a few minor points that should be addressed in a revised version however. Firstly, on the (lack of) biological activity of the NQS redox state, there is an earlier report from the lab of Sancar (JBC, 2008) claiming that the neutral radical form of DmCry does indeed display biological activity in cell culture systems. Another very recent study also proposes something along these lines (BaiK et al, PNAS, 2020, referenced as 57 in this manuscript). These studies should be more fully discussed, especially with respect to NSQ formation and biological role (if for no other reason than to prevent unnecessary confusion).

Another issue is the peptide tag plus spin probe placed at the C-terminal of the DmCry, the authors assume this will in no way perturb the system and have very nice modelling and structural data to back up this interpretation. However is there perhaps also evidence that fusions at the C-terminal domain do not affect DmCry biological activity in vivo in the literature? Could this tag perhaps affect the dark state conformation of the protein and alter its response? Possibly this could be a reason for the many instances of partial responses (docking/undocking) in this paper. Finally there is the more general question of a multiplicity of biological signaling mechanisms for DmCry, where C-terminal undocking may not always be required. The authors do indeed allude to this possibility (discussing ref 57), but could their discussion perhaps be expanded? For example, in the case of plant cryptochromes, there is a role for the C terminus in some responses but not for others, and this is thought to result from the fact that biologically relevant conformational changes can occur in the N-terminal domain as well as in the C-temrinal. Can the authors speculate a bit further on conformational changes in the N-terminal domain in DmCry, for example.

Finally, the authors should respond to a recent paper Kutta et al. , Phys Chem Chem Phys. 2018 Dec 7; 20(45): 28767–28776. which forwards the odd claim that DmCRY is a 'one-shot' photoreceptor that promptly loses its flavin every time it is illuminated (and then presumably just unfolds and degrades). Clearly this would provide a mechanism completely at odds to what is here presented (ie that reversible C terminal docking and undocking mediates biological activity depending on flavin redox state). It seems that the data in this currently submission, as well as that in many prior papers on which this submission has been based, will provide an appropriate response. This should however be made explicit in the discussion to provide greater clarity of the results.

Reviewer #3 (Remarks to the Author):

The authors address the general question of how the light-induced reduction of the flavin cofactor of cryptochromes may lead to conformational changes in the C-terminal extension (CCT). As a model, they investigated Drosophila cryptochrome, developing a highly sophisticated approach by spin labeling the CCT via a sortase-mediated ligation and applying DEER experiments for distance determination to the flavin radical. The key result is a semi-quantitative evaluation of the population of active/undocked to inactive/bound CCT in dependence of different mutations. This novel approach allowed the authors to obtain considerable new insight into the signaling pathway of Drosophila cryptochrome. The conclusions drawn on the role of the CCT undocking in TIM degradation but not CRY degradation are convincing.

1. The greater picture of cryptochrome mechanisms would be informative to include. The switch from an 'active' anion radical to an 'inactive' neutral radical should be discussed in the framework of the possible activity of dCRY in the fully reduced state and of other CRYs in their respective radical states. Is it valid to transfer knowledge from this study to other members of the CRY family? Please add the number of amino acids of the CCT to the abstract and to the introduction to clarify that dCRY as a model has an exceptionally short CCT in contrast to the more complex CCTs with >> 100 aa in the CRY family.

2. The L405E/C416N variant was designed to enable access to the bulk solvent. How does then the external pH influence the yield of neutral radical vs. anion radical? It needs to be mentioned in the main text that the L405E/C416N replacement shifts the maximum of absorbance of FADox in dCRY. In addition to the convincing UV/vis spectra of anion and neutral radical for L405E/C416N, such information should be provided for the triple mutants with histidine replacement as well.

3. The important fact is missing in the main text that the dCRY CCT was modified to contain the sortase recognition sequence. This fact needs to be critically discussed with respect to potential effects on the mobility and interaction of the CCT.

4. As a control for the fitting procedure, what are the percentages and error achieved by fitting two distances to the P(r) function of the wild-type light state (Figure 4b)? Please include in Table 2 for comparison.

5. Top and bottom panels of Figure 5 would better be swapped to provide the more logical association to dark state proxies and light states, respectively.

6. Some more details should be provided on the iLOV (plasmid, purification...) and a reference might be included to Christie et al. JBC 2012, 22295.

7. The green sphere included in Figures 1c and 4c is not mentioned or explained in the captions.

We thank the reviewers for their very useful and constructive critiques. We have performed new experiments and carried out new analyses to address their concerns. Additionally, we have made changes to the manuscript to improve clarity and better discuss key points, as they have recommended. Our detailed response follows:

Reviewer #1 (Remarks to the Author):

Summary
The goal of this study was to unravel the influence of the protonation state of the light induced FAD radical (protonated=NSQ versus anionic=ASQ) in Drosophila cryptochrome (dCry) with respect to the release of the CTT, and to elucidate the influence of the amino acid H378. A number of different variants have been produced and measured via pulsed EPR spectroscopy. The first main goal was to generate a variant that stabilize the NSQ and compare it with the WT (which forms an ASQ). This has been achieved by a L405E/C416N mutant. The variant indeed forms a NSQ, which was briefly analyzed by optical and EPR/ENDOR spectroscopy.

The labeling strategy by using sortaseA to covalently attach a spin label to the C-terminus is elegant and state of the art as it does not require any additional cysteine mutations within the dCRY protein. The subsequently performed DEER measurements are backed-up by a crystal structure of the dark state (dCry+Sortase-spin label), which provides precise information about the distance between the FAD and the SL. DEER analysis using dCry in its signaling state showed that the undocked CTT (=signaling state) has a ~40A distance with a broad distance distribution, whereas the docked CTT (=dark state) has a ~30A distance. The latter distance is almost identical to the one obtained from the crystal structure. Additional DEER experiments using three different H378 variants showed a different behavior of the three variants H378K/N/R with respect to the variants with H378. Whereas H378+ASQ shows CTT displacement, H378 variants seem to stabilize the CTT.

Conclusion:
Studying signal transduction processes of insect cryptochromes is a highly relevant issue and would merit the requirements for publication in "Nature Communications Biology". The finding that only the ASQ but not the NSQ redox state promotes CTT conformational changes is an important novel finding and could help to fully understand molecular details of signal propagation from the FAD chromophore to the surface of the cryptochromes protein. However, the influence of H378 still remains mainly unresolved, but its obvious that it has an important influence. In sum, I would recommend publication after major revision.

Specific comments:

1) ENDOR analysis

- Figure 2 legend: Please exchange: “The dCRY L405E/C416N variant has -peaks-“ with “hyperfine couplings” or with “shows frequencies of X MHz around the Proton Larmor frequency”.

We have updated the legend to read “The dCRY L405E/C416N variant has hyperfine coupling values at 5.0, 6.8, 10.8 and 25.3 MHz corresponding to the H6, H8, H1’ and H5 protons, respectively”.

- Table1 legend: The weak signal intensity of the H5 hyperfine coupling is mainly because of its large anisotropy. Moreover, the peak at 25.3MHz corresponds to one principal component of the hyperfine tensor. This should be clarified.

Thank you for this comment, it is true that the H5 hyperfine coupling has weak intensity owing to a wide range of factors including the large anisotropy of this hyperfine term. The choice of the ESR frequency (and spin tensor term excited) also affects the signal strength. We have added the following statement to the Table legend: “For EN, the peak at 25.3 MHz corresponding to the proton on the N5 nitrogen is weak due to variety of factors that include large anisotropy of the hyperfine tensor as well as the relatively weak power of the r.f. amplifier in that region.”

- the ENDOR data convincingly show that the FAD radical is protonated in the L405E/C416N variant. A discussion of the different proton hyperfine couplings, perhaps even with respect to other cryptochromes/photolyase data is missing.

Flavin ASQ and NSQ radicals have been characterized by ENDOR previously and there is little exceptional about these spectra for dCRY. We rather primarily use the ENDOR spectra to confirm these states. However, we agree that more some discussion, especially with reference to other papers in the field is warranted:
Hoang et al. PLOS Biology. 2008. (X-band ENDOR of dCRY in vivo and in vitro)
Bouly et al. Journal of Biological Chemistry. (X-band ENDOR of Arabidopsis cry1 in vivo and in vitro)
Kay et al. Biochemistry (1999) (X and W band ENDOR of E. coli DNA photolyase)
Lines 112-116:
"The hyperfine frequencies observed for dCRY WT agree well with previously reported values for the ASQ radical[14,40]. Additionally, the frequencies observed for dCRY L405E/C416N variant agree well with other NSQ forming cryptochromes such as *Synechocystis* species cryptochrome-DASH and *Arabidopsis thaliana* cryptochrome 1[14,40,41]. "

2) iLOV construct.
The DEER time trace hardly shows any modulation; therefore, the resulting distance distribution may contain substantial errors (see also the general comment about the analysis of the DEER traces). In addition, most LOV domains show significant (unspecific) dimerization at higher concentrations in vitro, which would result in a number of distances or a broader distance distribution. A control experiment containing the iLOV/FMN radical alone could clarify this issue.
As a more general question: Why is this sample included in this study as the crystal structure of dCry+Sortase-spin label already showed that the sortase reaction is successful? What is the purpose of this sample?

Although the paper is largely focused on the CTT of dCRY, we believe it is useful to demonstrate that the sortase labeling reaction and the flavin-nitroxide DEER experiments can be applied to other flavoprotein systems such as LOV domains. Flavin-nitroxide DEER is relatively rare and some of the technical details discussed in this paper will hopefully assist other users with applying this technique.

iLOV was specifically chosen to pilot this approach because iLOV is a small monomeric protein (~12 kDa) that forms an FMN rather than FAD radical. iLOV was developed to be a monomeric system [*Current Opinion in Chemical Biology* vol. 27 39–45 (2015)], thus there is no reason to expect substantial dimerization. Furthermore, the crystal structure of iLOV allows us to validate the distance between the C-terminus and the flavin moiety. Our results are in agreement with the distances expected from the crystal structure.

Although we expect no dimerization from iLOV, a DEER signal between flavin radicals would not interfere with our analysis. The temperature (~60 K) of the experiment substantially reduces the flavin-flavin DEER signal and the only signal observable is between flavin-nitroxide and nitroxide-nitroxide. Furthermore, the pump-probe frequencies are separated by 84 MHz, this large separation between the pump and probe frequencies also reduces the nitroxide-nitroxide DEER signal.

To clarify this point we have added:
Line 142-143: "The engineered iLOV protein chosen is relatively small (~12 kDa) and does not dimerize upon light irradiation[52]."

We believe that there are multiple reasons for low modulation with the iLOV sample. Mainly, we have observed that iLOV has low photoconversion efficiency in the absence of sacrificial electron donors (such as EDTA). Additionally, long illumination times that are necessary to achieve photoconversion lead to partial reduction of the nitroxide probe. Furthermore, flexible domains such as the LPGTGGGG linker lead to low modulation.

3) Deer analysis
- Most samples contain a significant amount of "free" spin label (sharp signals in Supporting Fig.10 and 11). I guess that these spins add significantly to the DEER background signal and may obscure any wanted modulations. Perhaps, a second desalting step directly before light irradiation would be helpful.

We have done several steps to reduce the amount of free spin label in the DEER measurements. In our approach the SORTC-SL (GGGGC-SL) peptide is pre-purified with TLC, there is no excess MTSL. Furthermore, we add only a slight molar excess (~5-10%) of the SORTC-SL peptide for the dCRY/Sortase reaction. Labelled dCRY is then separated from excess Sortase and SORTC-SL with size exclusion chromatography (S200 analytical column) before being buffer exchanged into D20/d8-glycerol for DEER measurements.

The sharp signals in Fig S10/S11 are indicative of a nitroxide with motion on the 0.1-1 ns timescale as compared to free spin label (0.010 ns). These features are more pronounced with the H378R, H378R and H378N mutants which is in agreement with the DEER results showing that these mutations promote CTT release in the dark.

The presence of background in DEER spectra has been well studied. Multiple factors contribute to the DEER background in our samples, primarily

1. Multiple spin effect: In the concentration regime we are working in (0.1 – 1 mM), there is a small amount of intermolecular spin effect, i.e., each flavin-nitroxide radical pair on one dCRY is affected by the presence of radical pairs on other dCRY molecules.
2. Presence of single spins: The photoconversion of dCRY FAD^{ox} to the $FAD^{ASQ/NSQ}$ state is not always 100% leading to unpaired SL. Additionally, the partial reduction of the nitroxide radical by intense blue light leads to unpaired $FAD^{ASQ/NSQ}$. These single spins do not contribute to the DEER oscillations but give us rise to an exponential background.

Given the sample preparation, we therefore attribute the presence of DEER background to these other contributing factors and not the presence of free spin label.

ELDOR time traces (Figure 4 and S8): Unfortunately, I think that the DEER analysis of the H378 variants is slightly overstated and should be reevaluated carefully.

We have performed the DEER analysis as rigorously as possible using two methods –a model based gaussian fit (DD) and a model free fit (SVD, [Srivastava and Freed. J. Phys. Chem. Lett. 2017, 8, 22, 5648–5655]). Analysis with both methods (discussed further below) yield similar results and both support the notion that the H378 mutants lead to CTT undocking in the dark.

Both the text and SI have been updated to reflect the choice of data analysis.

A few examples for this statement:
(i) the DEER analysis of WT and L405E/C416N only show one distance. The analysis of H378R (Fig. 5) however results in only 80% open state (like the WT) although the experimental datasets are almost identical to the one obtained from the WT (Fig. S8). I would recommend to analyze the WT (with FADox), which should give no distance distribution but a background signal, and the WT (FAD ASQ) using the same model to evaluate the quality of the fits.

(ii) The datasets of the three H378K/L405E/C416N and H378R are very similar (Fig. S8), the percentage of open/closed state are quite different (Fig. 5).

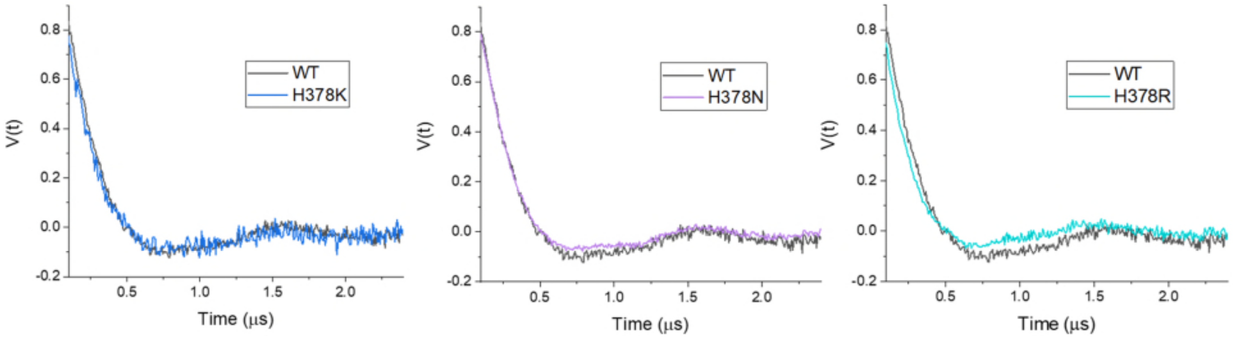

Figure 1: Background subtracted time domain trace of WT and H378 variants.

The data shown originally in the supplement was not background subtracted which makes it difficult to observe the relevant differences. The two time-domain traces for (dCRY WT and dCRY H378R) appear very similar, but have features that are evident in the P(r) domain. As seen above (Response Figure 1), the two traces for WT and H378R have small but significantly different exponential decays between 0-0.5 μs. These differences in the time domain leads to the nearly 20% difference in the population of the docked state. We have now updated the supplement (Figure S10) to reflect the background subtracted time domain data.

(iii) The signal/noise ratio of sample H378N/L405E/C416N is lower than in the other samples. Nevertheless, the error of the fit is considered to be the lowest of all samples (Table 2).

The apparent mismatch between signal-to-noise (SNR) in the time domain and fit error is because the error values are not absolute and are normalized by the SNR of the experiment. In a model based fit, it is important to differentiate error from the fit and the error from the SNR of the experiment. We have now added a clarification to the methods section to reflect these points.
"From DD, in addition to the distance reconstructions and population values, we also obtained the noise corrected error (χ_v^2) values[75], $\chi_v^2 = \frac{1}{N-q}\sum_{i=1}^{N}\frac{[V(t_i)-F(t_i)]^2}{s_i^2}$, where V(t) and F(t) are the experimental and the fit data respectively, N is number of points, q is number of variables changed and s_i is estimated noise level for the ith point. These error values are indicative of fit between the experimental data and the distance reconstruction, values below 2 are good fits. The details of DD fitting and variables have been documented elsewhere[55,75,76]."

We have also added the percentage errors to the variances of the undocked state in Table 2 and better explained these errors in the legend:

"**Table 2:** Percentage of the undocked state observed after light irradiation for H378 variants along with their variance. Residual X^2 error estimates are determined in DD and indicate the robustness of fit between the experimental data and the distance reconstruction. A residual X^2 error value in the range of 2 or lower is indicative of good fit. The variance in the population of the undocked state is also given. The H378K/L405E/C416N variant (*) has relatively high error and is discussed in **Figure S16**."

(iv) no simulated spectra are presented. It is however quite crucial to show (i) the spectra after baseline subtraction and (ii) the subsequent processing of the spectra. It would be particularly important to overlay the experimental spectra and their respective simulations so that the quality of the fit can be inspected by the reader

Perhaps, repeating the analysis with a different program without any model (e.g., DEER analysis or other) would help to strengthen the robustness of the fits. Another possibility would be to attenuate the absolute values of the two distance distributions (Table 2) and only provide approximate values as the main statement (H378 variants stabilize the CTT even in the signaling state) remains the same anyway.

We now provide fits to the time domain data obtained with both DD and SVD (below). Although the SVD method provides a better fit for all samples (Response Figure 2, 3), the P(r)'s obtained make quantification of docked/undocked states less straightforward. Particularly, SVD method (and Tikhonov regularization carried out in DeerAnalysis) derived P(r)'s is susceptible to corruption by noise. Further, this approach will require choosing different cutoffs for the singular values (and lambda for Tikhonov regularization), the choice of this cutoff will depend on the SNR in the time domain data. As different time domain datasets have different SNRs and therefore different cutoffs (and different errors), we decided against fitting the H378 variants with the WT and L405E/C416N variant in the distance distribution (P(r)) domain. However, qualitative observation of the H378 variants using the SVD methods shows that the general trend observed in the DD method is followed.

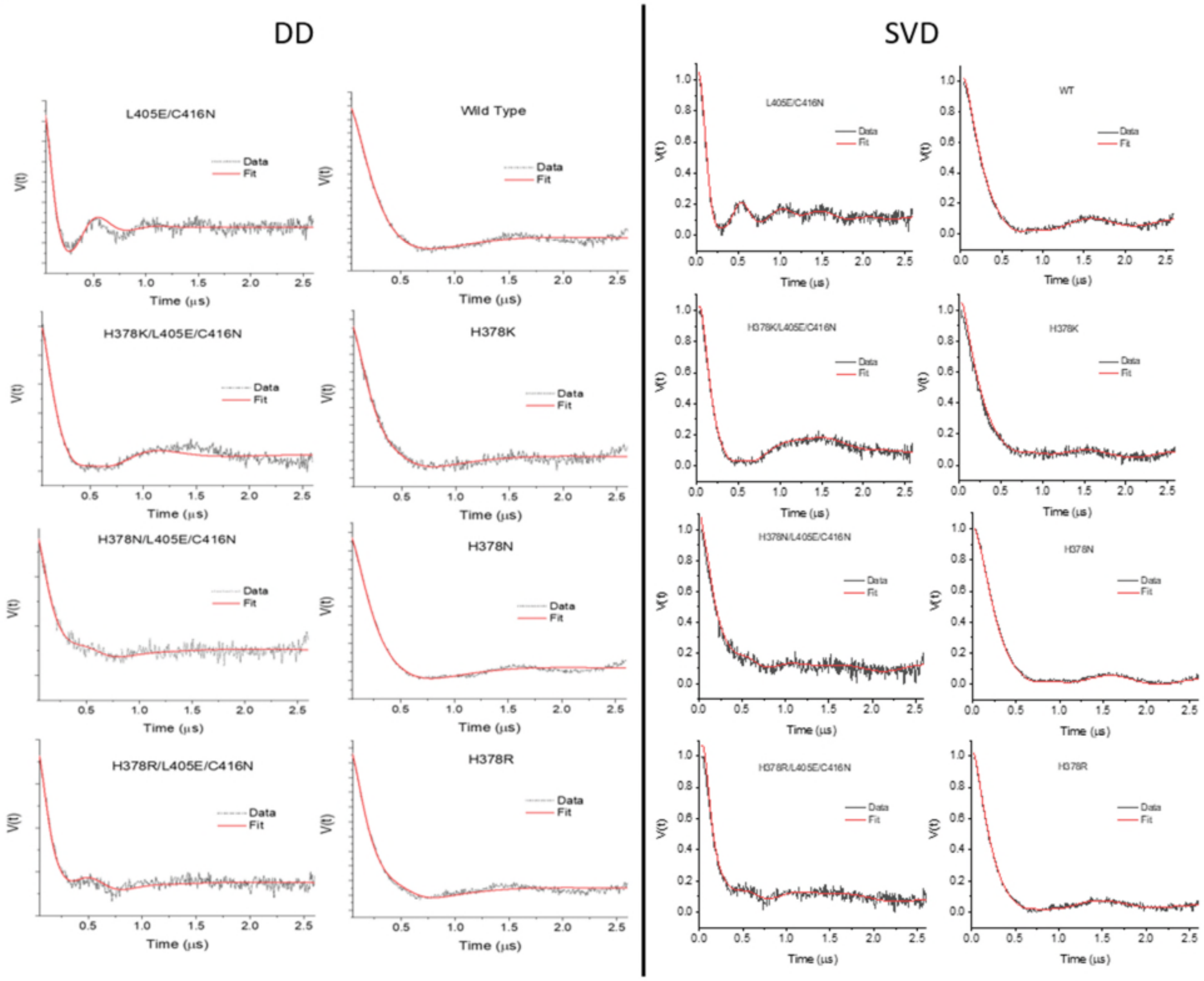

Figure 2: The time-domain fits performed in DD and SVD compared to experimental spectra. The error from the fits have been tabulated in Table 2 in the main text.

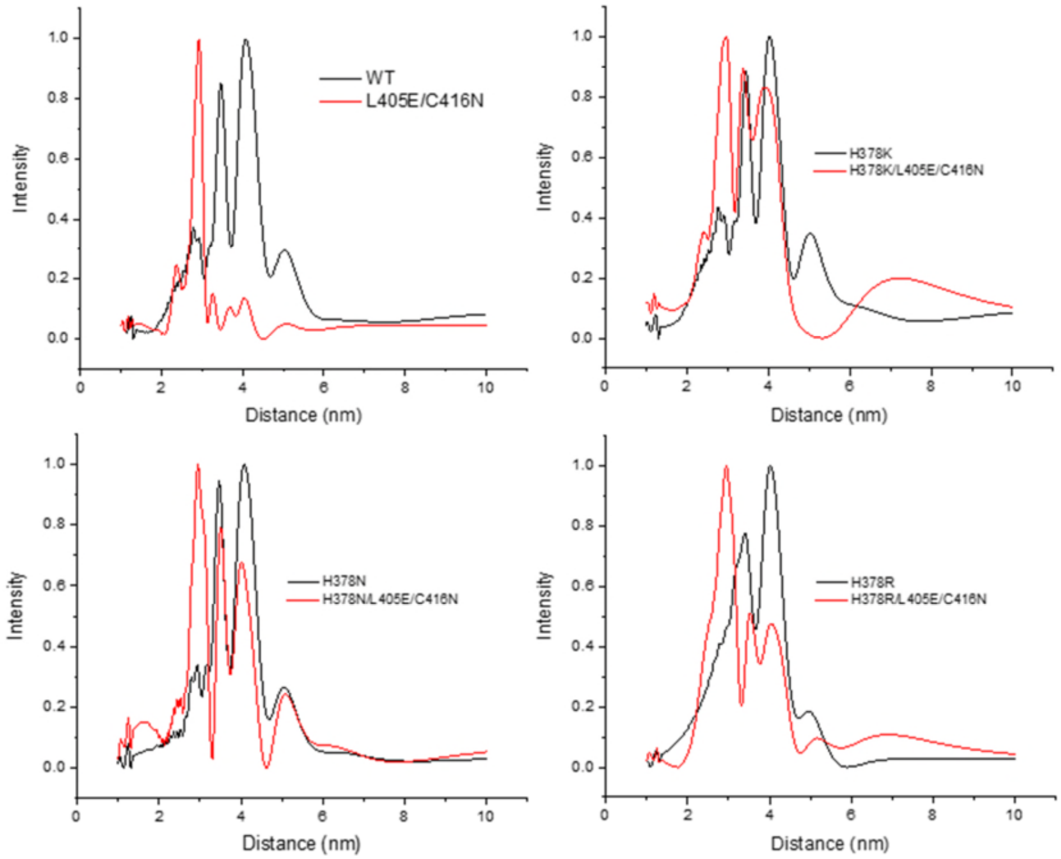

Figure 3. Distance distributions obtained using SVD for each mutant.

To enable quantification of the H378 variant distributions by a method other than DD, we directly fit the H378 variant time domain data as a linear combination of both the WT (undocked) and C416N/L405E (docked) time domain spectra (below). We have fit the background subtracted time domain (TD) data for the H378 single and triple variants using the TD traces of the dCRY wild type and L405E/C416N variant. We obtained the best fit population values by varying the population of the docked states and minimizing the root mean square error between the raw data and the fitted trace. This "model-free" method does not include other parameters and therefore directly models the variants with the time domain traces themselves. These results are reported in Response Figure 4 and Table 1 below with values obtained using DD in parentheses. We report the same figures and data in Figure S14 and Table S2.

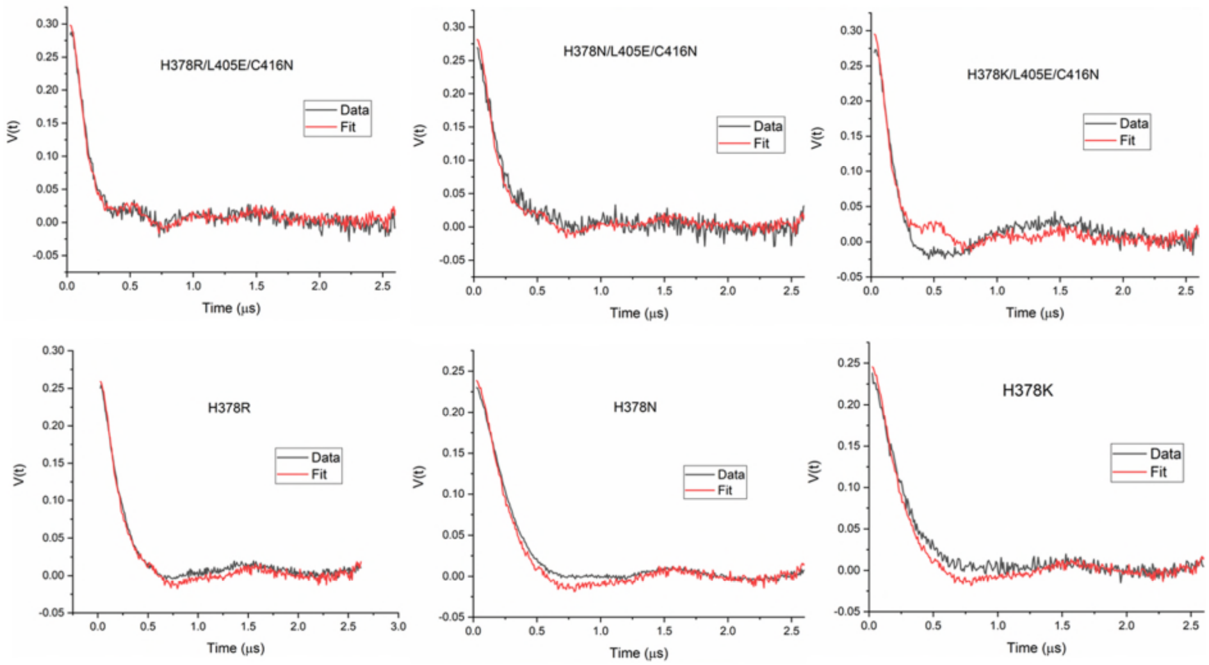

Figure 4. Direct fitting in the time domain overlaid with experimental data.

Table 1: Population of the CTT in the docked state obtained by fitting directly in the time domain. The values presented in the parenthesis are the value obtained in DD by performing a restrained two component fit.

	R378	N378	K378
**E405/N416**	47 (47)	63 (63)	50 (57)
**L405/C416**	81 (81)	96 (95)	91 (92)

The values obtained from this two component fit agree well with the two component fit performed using DD. Thus, we are confident about the analysis performed and believe the populations tabulated are accurate. Furthermore, the DD approach that fits the DEER data in the time domain is better suited for fitting multiple components than the SVD or Tikhonov regularization based methods. Note: the DD approach is not universal and has limitations, particularly, it can be prone to trapping in local minima (even though the most recent versions of the fitting algorithms are improved in this respect) and distance distributions needn't always assume a Gaussian distribution (A Gaussian fit is a good first order approximation).

We chose to use an ESR based approach because of its relative quantitative accuracy, the fitting of gaussians to the DEER reconstructed data has been previously well established, [J Mol Biol. 427, 867–886 (2015)] and [Nat. Struc. Molec Bio. 20, 215-221 (2013)]. However, any model-based fitting approach has its limitations. We have attempted to limit bias by using as objective an approach as possible.

4) Interpretation

- H378 variants: I think that another possibility has to be discussed: Triple variants as used in this study are likely to modify the surrounding between FAD and the CTT and could lead to unwanted conformational changes at the CTT and/or could modify signal propagation from the FAD to the CTT. Although this is an unavoidable problem when working with variants, it should at least be stated.

Thank you for this comment. A crystal structure of the dCRY L405E/C416N (in tan) variant that we have determined overlaid with dCRY WT (white) shows no large structural changes upon addition of the L405E/C416N mutations (Response Figure 5). We therefore hypothesize that the L405E/C416N

mutations help tune the flavin redox environment by only increasing FAD solvent accessibility. We plan on reporting the crystal structure of L405E/C416N along with a more detailed characterization of its properties in a future study, and would prefer not to include it here. The L405E/C416N residues are located on the distal end from the CTT with the FAD sandwiched in-between. We therefore believe that the L405 and C416 variants will not directly affect the CTT. We have also updated the Fig, 1B of the main text to reflect this point and it is now discussed in the legend to Figure 1.

[Figure 5 redacted]

- While the sortase reaction is elegant and has significant advantages (as pointed out in the text), it has also a downside as only the signaling state can be investigated. In this case, the amount of CTT release in the H378 variants under dark-state conditions remain unclear. This should be mentioned somewhere in the Discussion section.

Thank you for the comment, in our initial submission we did make an attempt to make this point clear. Our rationale in designing the C416N/L405E variants was to use these proteins conformational representativies of the dark state (a proxy). We present cw-ESR data of dCRY H378 variants in the dark state (Fig S10), this data indicates that the C-terminus spin label is significantly more flexible in the H378R,K,N variants as would be expected when the CTT is docked. The cw-ESR measured in the dark and the DEER measured in the light combined supports the strength of the sortase based labeling technique.

To better clarify we changed lines 80-82:
“We thus reasoned that dCRY containing the neutral semiquinone (NSQ) would still bind the CTT and thereby act as a functional mimic of the dark-state.”

5) Minor comments (without specific order)
- are the samples prepared under aerobic or anaerobic conditions?
The samples were measured under aerobic conditions. This has been added to the methods section.

- line 474: please remove brackets
- Figure 2 legend: Please exchange H8 with H8alpha
We have updated the text with these changes.

- The alignment and the abbreviations of the variants shown in Fig. 5 and Fig. S8 are very different, which does not support its readability and inhibit direct a comparison. Please adjust.
Thank you, we have updated Fig. S8 for consistency.

- line 335: The sentence is misleading as all presented data are recorded in the signaling state.
“Signaling” state for CRYs typically involves CTT release leading to Timeless degradation. (The new Fig.S7 demonstrates that CRY without the CTT binds Timeless constitutively) Our evidence suggests that the NSQ-formed L405E/C416N variant is not the signaling state. It is presumed that further reduction of the NSQ to the hydroquinone in fact could correspond to a “signaling” state, therefore we consider that the L405E/C416N variants correlate to a conformational “dark” state. To avoid confusion, we changed it to “docked” and “undocked” state instead of the “on” and “off” states.

We have also added a brief discussion of the role the hydroquinone may play in some recent observations about the red-light sensitivity of dCRY.

Lines 427-431:
“It is notable that the ability of dCRY to modulate neuronal firing frequency has a small red-light dependence[67]. As the NSQ would be the only flavin species to absorb at these wavelengths, the red-light effect may involve conversion of a small pool of inactive NSQ to active fully-reduced $FADH^{-}$, which, like the ASQ, would carry a negative charge.”

Reviewer #2 (Remarks to the Author):
This manuscript provides for the first time a complete sequence of events that lead from light-triggered redox changes at the flavin pocket in Dcry to unfolding of the C-terminal domain that triggers biological activation. To do so, the authors have used an elegant, technically flawless spin labelling protocol that specifically attaches a single nitroxide spin probe cleanly at the C-terminal end of the dCRY protein. As a reference spin probe, these authors used the flavin radical (ASQ) for the light – induced dCRY conformation. What is particularly impressive is their characterisation and use of an engineered dCRY variant producing the neutral SQ radical as a dark - state proxy, as this NSQ radical does not allow undocking of the C-terminal domain in response to light and thereby is not biologically active. Pulse dipolar electron-spin resonance spectroscopy allowed identification of significant conformational change and increased motion of the C-terminal domain as a consequence of illumination in the ASQ but not NSQ state, consistent with biological activity. Furthermore these authors performed elegant studies of C-terminal conformational change on a series of mutations at the His378 position, which prior studies had extensively documented in terms of biological activity and impact on flavin redox chemistry. Since all of the results obtained from this study are internally consistent, concur with molecular dynamic simulations, and correspond to or are corroborated by prior studies of biological activation by this receptor, the paper is timely and will be warmly welcomed in a field that has been characterized by much past confusion due to conflicting methodology and differing, largely indirect approaches.

We thank the reviewers for their comments and appreciate that they consider that our results will be of interest to the community.

There are a few minor points that should be addressed in a revised version however. Firstly, on the (lack of) biological activity of the NQS redox state, there is an earlier report from the lab of Sancar (JBC, 2008) claiming that the neutral radical form of DmCry does indeed display biological activity in cell culture systems. Another very recent study also proposes something along these lines (BaiK et al, PNAS, 2020, referenced as 57 in this manuscript). These studies should be more fully discussed, especially with respect to NSQ formation and biological role (if for no other reason than to prevent unnecessary confusion).

We have updated the text to discuss the role of NSQ formation in dCRY signaling.

Lines 428-431
"It is notable that the ability of dCRY to modulate neuronal firing frequency has a small red-light dependence[67]. As the NSQ would be the only flavin species to absorb at these wavelengths, the red-light effect may involve conversion of a small pool of inactive NSQ to active fully-reduced $FADH^{-}$, which, like the ASQ, would carry a negative charge."

Another issue is the peptide tag plus spin probe placed at the C-terminal of the DmCry, the authors assume this will in no way perturb the system and have very nice modelling and structural data to back up this interpretation. However is there perhaps also evidence that fusions at the C-terminal domain do not affect DmCry biological activity in vivo in the literature? Could this tag perhaps affect the dark state conformation of the protein and alter its response? Possibly this could be a reason for the many instances of partial responses (docking/undocking) in this paper.

Thank you for this comment. We are working with the assumption that "the sort-tag is present in all our variants, so any changes we see can be directly attributed to the mutations and not the sort-tag itself". Given that the peptide tag (9aa) is present on both the docked (L405E/C416N) and undocked (WT) samples and drastic changes in P(r) are observed between the two, we conclude that that the flavin radical state (neutral or anionic) is the major factor in determining the CTT conformation. Furthermore, in the crystal structure of the WT protein containing the sortase tag, we don't observe any perturbations to the CTT in comparison with the WT protein lacking the SORT-C-SL tag. In fact, sortase linker is pointing away from the CTT and PHR and is not well ordered. Additionally, the MD simulations in this paper have been carried out without the SORT-C-SL tag, and since our ESR data is relatively consistent with these simulations we believe addition of the peptide tag does not greatly affect CTT release.

Nonetheless, the reviewer raises a valid criticism with respect to possible perturbations induced by the tag. As such, we now present a new set of experiments conducted to probe the consequences of introducing the tag on dCRY activity. In these experiments we make use of a fluorescence detection assay to allow quantification of TIM levels and dCRY-TIM interactions for proteins expressed in insect cells. During the binding experiments steps are taken to prevent light-mediated degradation of either species so that the binding interaction can be isolated. Unfortunately, these experiments cannot be carried out with the nitroxide probe itself, because it is unstable in the reducing environment of the cell and TIM cannot be produced in a functional form outside of the cell. Instead, we have replaced the LPGTGGGGC-SL tag with LPGTGGGGH tag, in which the histidine group mimics the size of the spin labeled cysteine residue. Overall, the experiments show that introduction of the sortag still allows light dependent TIM degradation by dCRY and light-induced binding of dCRY to TIM (Figure S7). In the case of TIM degradation activity, sort-tagged dCRY is similar to WT, expecially considering that the tagged version appears to be expressed at a slightly lower level than WT and TIM degradation depends on overall dCRY expression levels, in addition to light (note CRY-ΔCTT Figure S7). It is true that the magnitude of the binding effect is reduced somewhat for sort-tagged dCRY compared to WT (Figure S7). It is interesting that the tag appears to interfere somewhat with TIM binding and suggests that the "undocked" conformation of the CTT may localize close to the TIM interaction region. Thus, the LPGTGGGGC-SL tag may not directly influence the undocking of the CTT but does affect dCRY binding to Timeless in the light. The reduced binding does not seem to greatly effect cellular activity under these conditions.

Lines 181-192:
"We implemented a quantitative fluorescence-based assay to evaluate the light-induced degradation of TIM by sort-tagged dCRY in insect cells. The nitroxide spin-label is unstable in vivo, and thus, we tested a SORTC tag that had the terminal Cys residue replaced by an His to mimic the size of the nitroxide moiety (LPGTGGGGH). Sort-tagged dCRY effectively degrades TIM in the light with a similar activity as WT (Figure S7). Because TIM degradation by dCRY depends on expression level, which differs among the variants, to aid comparisons we also measured light-induced binding of dCRY to TIM. As expected, WT dCRY binds TIM preferentially in the light, whereas a dCRY-ΔCTT variant binds TIM equally in light and dark. Sort-tagged dCRY also binds TIM considerably better in the light, but the difference is less than with WT. The additional residues of the SORTC-SL tag may interfere with TIM binding to some extent, but TIM degradation is not greatly affected (Figure S7)."

Finally, there is the more general question of a multiplicity of biological signaling mechanisms for DmCry, where C-terminal undocking may not always be required. The authors do indeed allude to this possibility (discussing ref 57), but could their discussion perhaps be expanded? For example, in the case of plant cryptochromes, there is a role for the C terminus in some responses but not for others, and this is thought to result from the fact that biologically relevant conformational changes can occur in the N-terminal domain as well as in the C-temrinal. Can the authors speculate a bit further on conformational changes in the N-terminal domain in DmCry, for example.

The reviewer makes a good suggestion for placing our work in a broader context. We have included discussion of the role that CCE signaling plays in the plant and mammalian CRYs and how these other systems may relate to interactions observed for dCRY. With respect to the clock function and TIM engagement, the ΔCTT variant binds TIM constitutively, which we emphasize quantitatively in our new pull-down experiments (Figure S7). Thus, the PHR is the primary binding determinant for TIM. However, it is also interesting that the SORT-tag reduces TIM binding, which suggests that the undocked CTT is not far from the TIM interaction surface and could possibily participate in binding. Because the crystal structure shows that that the SORT-tag is largely disordered, we don't think the reduction in TIM binding is caused by enhanced interactions between the SORT-tag and the PHR. Our original proteolytic mapping experiments did indicate conformational changes additional to the CTT (Anand et al. 2013), but the regions affected largely surrounded the CTT. It will be interesting to observe the N-terminal domain changes in dCRY upon light activation, but we submit that such studies are beyond the scope of the current paper.

See new first paragraph of the discussion – lines 336-359:
"Following conformational changes in photoreceptor proteins is challenging because light-adapted states are often more dynamic and heterogeneous than their more stable dark-state precursors[2,58]. It has been long recognized that the CCEs have important roles in function that become unmasked by light activations. For the Arabidopsis thaliana CRY1 and CRY2 proteins, expression of the CCEs alone produces constitutive growth phenotypes[59]. Indeed, recent work shows that a specific sequence in the CRY2 CCE mediates interactions with ubiquitin ligase SPA/COP1[60]. The plant CRY CCEs are largely disordered in isolation but structure upon interaction with the PHRs22. In the case of plant CRY1, light-induced formation of a negative charge in the active center involving an aspartate residue that donates a proton to the flavin may promote release of the CCE and subsequent unveiling of its target sequences[23,61]. However, not all interaction partners of the plant CRYs require the CCE. For example, plant CRY2 binds the basic helix-loop-helix proteins CIB1 in a light dependent manner, independent of the CCE62. The CCEs of mammalian CRYs (mCRYs) have similar functions in molecular recognition, but their conformational states are not linked to light-activation. For example, a region of the mCRY1 coded by exon11 mediates with Clock:Bmal1 and naturally occurring genetic polymorphisms that affect this region alter human sleep behavior[21]. In addition, isozyme specificity of small-molecule inhibitors of mCRYs that target the cofactor binding site depends on the respective CCEs, which again suggests that the CCEs interact with the PHRs[63]. In the case of dCRY, the CTT has been long recognized to be an autoinhibitory element that gates interaction of the PHR with TIM[16–18], as shown by the light independent binding of dCRYΔCTT (Figure S7). Photoinduced conformational changes in dCRY have been generally monitored through proteolytic susceptibility and small-angle x-ray scattering[15,26,31] and MD simulations have provided an atomistic description of CTT motion[13,24,31]. However, direct experimental tracking of CTT structure and dynamics in the light state have been lacking."

Finally, the authors should respond to a recent paper Kutta et al. , Phys Chem Chem Phys. 2018 Dec 7; 20(45): 28767–28776. which forwards the odd claim that DmCRY is a 'one-shot' photoreceptor that promptly loses its flavin every time it is illuminated (and then presumably just unfolds and degrades). Clearly this would provide a mechanism completely at odds to what is here presented (ie that reversible C terminal docking and undocking mediates biological activity depending on flavin redox state). It seems that the data in this currently submission, as well as that in many prior papers on which this submission has been based, will provide an appropriate response. This should however be made explicit in the discussion to provide greater clarity of the results.

We have previously shown that light induced conformational changes in dCRY are reversible, and can be cycled (Anand et al PNAS 2013). In our current experiments, we observe that dCRY can undergo repeated reduction-oxidation cycles, i.e., reduction with blue light leads to formation of the SQ radical, which in the dark (with oxygen) returns to the ground state FAD(ox). This FAD(ox) can then be re-reduced to form the SQ radical with nearly identical kinetics. If the integrity of dCRY were compromised after photoactivation and/or dCRY was intrinsically designed to be a 'one-shot' photoreceptor than this sort of reduction-oxidation cycles would not be feasible. We have added an additional experiment to demonstrate that the CTT undocking can be repeated. Although we cannot rule out some changes to the protein upon light excitation, loss of the flavin is not an obligate component of the photocycle. With respect to the Kutta work, which we now discuss in more detail, even there, dCRY recovers to ~94% on each photocycle (see Fig. 4e,f in Kutta et al). Given that those experiments were done in the absence of reductant, it is not surprising that there would be some irreversible chemical change to the Trp triad during repeated photoexcitation. Such changes could conceivably lead to flavin loss, but we note that flavin loss was largely interpreted from changes in UV absorbance (lambda < 400 nm), and under the conditions used dCRY, oxidatively aggregates during repeated photocycles (now shown in supp. Fig S9, S10), such aggregation could also contribute to the changes in spectra observed.

Lines 216-228:
"Next, we probed if dCRY WT undergoes reversible reduction-oxidation cycles; i.e., after a photoreduction cycle of FAD^{ox} to the SQ radical followed by reoxidation back to FAD^{ox} in the dark, does the CTT release in a second cycle of photoreduction? Earlier UV-Vis and proteolytic protection studies reveal that the dCRY CTT undergoes reversible undocking in light upon formation of the ASQ radical and re-docking in

the dark upon recovery to the FAD^{ox} state[15]. Furthermore, CTT released by chemical reduction could be recovered and re-released with light[15] However, we wondered whether subsequent undocking produced conformations similar to those of the initial cycle. Indeed, 4P-DEER derived distance distributions of dCRY WT subjected to reduction-oxidation-reduction conditions are similar to those of the primary photoreduction (**Figure S9**), Thus, dCRY is relatively stable to photoreduction followed by reoxidation and the photocycle does not require flavin cofactor release or irreversible unfolding. We do note that in the absence of reductants (required to preserve the nitroxide moiety), dCRY undergoes some oxidative aggregation during recovery (**Figure S10**). ”

Lines 422-427:
“Recent studies indicate that under certain conditions repeated photoactivation of purified dCRY damages the Trp triad, which alters the photoreduction mechanism and may result in flavin loss[24]. Nonetheless, the majority of the protein (> 90%) recovers on each photocycle, and some loss of activity is unsurprising given that in vitro dCRY oxidatively aggregates in the absence of reductants. The DEER experiments also indicate that flavin loss is not an obligatory step of the photocycle and dCRY can repeatedly release the CTT during cycles of light activation.”

Reviewer #3 (Remarks to the Author):

The authors address the general question of how the light-induced reduction of the flavin cofactor of cryptochromes may lead to conformational changes in the C-terminal extension (CCT). As a model, they investigated Drosophila cryptochrome, developing a highly sophisticated approach by spin labeling the CCT via a sortase-mediated ligation and applying DEER experiments for distance determination to the flavin radical. The key result is a semi-quantitative evaluation of the population of active/undocked to inactive/bound CCT in dependence of different mutations. This novel approach allowed the authors to obtain considerable new insight into the signaling pathway of Drosophila cryptochrome. The conclusions drawn on the role of the CCT undocking in TIM degradation but not CRY degradation are convincing.

1. The greater picture of cryptochrome mechanisms would be informative to include. The switch from an 'active' anion radical to an 'inactive' neutral radical should be discussed in the framework of the possible activity of dCRY in the fully reduced state and of other CRYs in their respective radical states. Is it valid to transfer knowledge from this study to other members of the CRY family? Please add the number of amino acids of the CCT to the abstract and to the introduction to clarify that dCRY as a model has an exceptionally short CCT in contrast to the more complex CCTs with >> 100 aa in the CRY family.

In the introduction we have explicitly defined the CRY C-terminal Extension (CCE) and distinguished it from the CTT tail helix, including the relevant lengths.
Lines 22-26:
"Structurally, all CRYs contain both a conserved photolyase homology region (PHR) with the capacity to bind flavin adenine dinucleotide (FAD) and a CRY C-terminal extension (CCE) of variable length and sequence that often regulates interactions with other proteins[6–10]. In type I insect CRYs the 23-residue CCE ends in a highly conserved 10-residue helical C-terminal tail (CTT) that inserts into the flavin binding pocket [6,7]."

We have expanded the discussion to relate and compare the role of the CCE in other cryptochromes. Although there are likely similarities to the mechanism of CCE conformational change in the other systems, given that the CCEs are unique to each type of CRY the mechanisms also likely differ.

See new first paragraph of the discussion – lines 336-359 (and above).

2. The L405E/C416N variant was designed to enable access to the bulk solvent. How does then the external pH influence the yield of neutral radical vs. anion radical? It needs to be mentioned in the main text that the L405E/C416N replacement shifts the maximum of absorbance of FADox in dCRY. In addition to the convincing UV/vis spectra of anion and neutral radical for L405E/C416N, such information should be provided for the triple mutants with histidine replacement as well.

In all the pH ranges we have worked with 6.5-9.0 (see figure below), we observe that the dCRY L405E/C416N variant consistently forms the NSQ state upon irradiation. However, at pH 9.0 we observed initial ASQ formation that slowly converts into the NSQ radical, even with recovery under no irradiation. At pH 9 we had difficulty observing consistent results due to aggregation and low protein solubility.

We have observed similar such kinetics for the NSQ formation with other L405 variants. We do intend to explore other such variants in a future study.

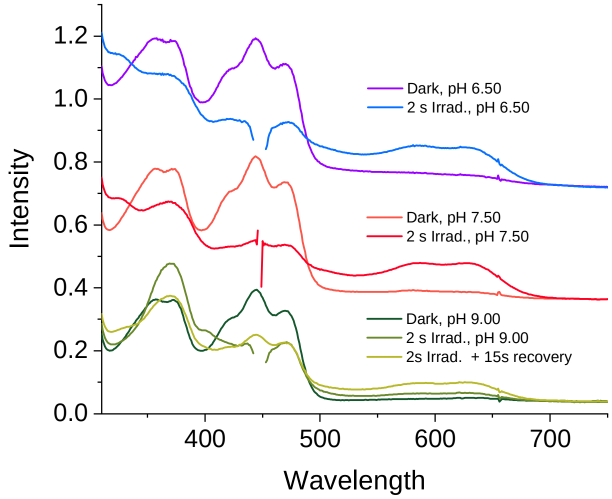

We have modified the text to mention this ~2 nm blue shift of the FADox maxima. It is conceivable that changing the solvent accessibility and the hydrogen bonding partner of the N^5 FAD group leads to this blue shift. We plan to examine this effect further in a future study.

We have added UV-vis spectra of the mutants as Figure S12.

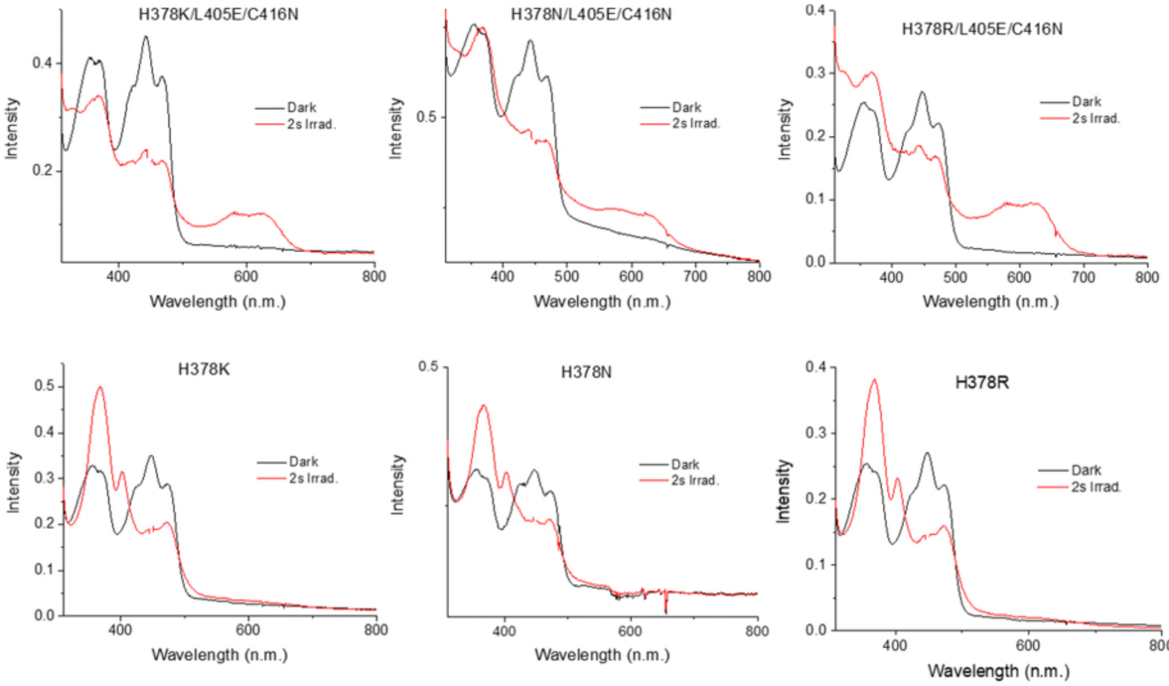

3. The important fact is missing in the main text that the dCRY CCT was modified to contain the sortase recognition sequence. This fact needs to be critically discussed with respect to potential effects on the mobility and interaction of the CCT.
Thank you for this comment, we have addressed this point in detail with respect to reviewer's 1 and 2 comments and have added the new insect cell dCRY:TIM interaction experiments to provide greater functional context for the modified proteins (Figure S7).

4. As a control for the fitting procedure, what are the percentages and error achieved by fitting two distances to the P(r) function of the wild-type light state (Figure 4b)? Please include in Table 2 for comparison.
The presence of the 2nd component, i.e., the docked state, does not significantly improve the fit for the WT ASQ form. We observe only 1% of the docked state; as this is within the error margin of the experiment we conclude that the docked state is not significantly occupied in irradiated WT.

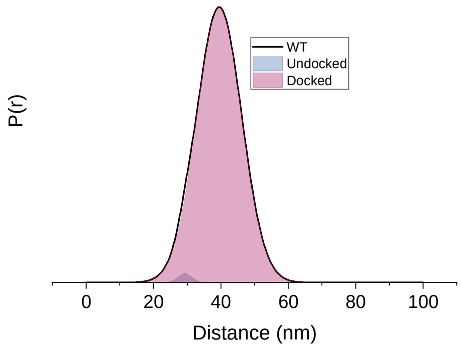

5. Top and bottom panels of Figure 5 would better be swapped to provide the more logical association to dark state proxies and light states, respectively.
Thank you, we have updated this figure as suggested.

6. Some more details should be provided on the iLOV (plasmid, purification...) and a reference might be included to Christie et al. JBC 2012, 22295.
Good point, we have added additional details in the methods section as well as references to John Christie's work on iLOV.

7. The green sphere included in Figures 1c and 4c is not mentioned or explained in the captions.
The green spheres are magnesium associated with the FAD, we have updated the figure captions to denote them.

Reviewer #1 (Remarks to the Author):

Dear authors,

Basically all my comments and suggestions were answered in detail and completely.
I would very much appreciate if the additional evaluations and illustrations, which were produced as answers to my comments, are published as Supporting Figures. This applies in particular to the comparison of DD and SVD fits (response Figure 2/3).

In summary, I support the acceptance of the publication

Reviewer #2 (Remarks to the Author):

The authors have fully addressed all of my concerns.

Reviewer #3 (Remarks to the Author):

The authors have carefully revised the manuscript and have met all my concerns. I only request that prior to publication the newly introduced sentence on line 344 is revised: '...light-induced formation of a negative charge in the active center involving an aspartate residue that donates a proton to the flavin may promote release of the CCE and subsequent unveiling of its target sequences23,61.' Reference 23 hypothesizes that ATP is expelled by the negative charge, which is a profound difference in mechanism.
